# Insights Into the Immune Response of the Black Soldier Fly Larvae to Bacteria

**DOI:** 10.3389/fimmu.2021.745160

**Published:** 2021-11-18

**Authors:** Daniele Bruno, Aurora Montali, Maristella Mastore, Maurizio Francesco Brivio, Amr Mohamed, Ling Tian, Annalisa Grimaldi, Morena Casartelli, Gianluca Tettamanti

**Affiliations:** ^1^ Laboratory of Invertebrate Biology, Department of Biotechnology and Life Sciences, University of Insubria, Varese, Italy; ^2^ Laboratory of Comparative Immunology, Department of Theoretical and Applied Sciences, University of Insubria, Varese, Italy; ^3^ Laboratory of Insect Biochemistry and Molecular Sciences, Department of Entomology, Faculty of Science, Cairo University, Giza, Egypt; ^4^ Guangdong Provincial Key Laboratory of Agro-Animal Genomics and Molecular Breeding, Guangdong Provincial Sericulture and Mulberry Engineering Research Center, College of Animal Science, South China Agricultural University, Guangzhou, China; ^5^ Laboratory of Insect Physiology and Biotechnology, Department of Biosciences, University of Milano, Milan, Italy; ^6^ Interuniversity Center for Studies on Bioinspired Agro-Environmental Technology (BAT Center), University of Napoli Federico II, Naples, Italy

**Keywords:** cellular response, hemocytes, *Hermetia illucens*, humoral response, immune system

## Abstract

In insects, a complex and effective immune system that can be rapidly activated by a plethora of stimuli has evolved. Although the main cellular and humoral mechanisms and their activation pathways are highly conserved across insects, the timing and the efficacy of triggered immune responses can differ among different species. In this scenario, an insect deserving particular attention is the black soldier fly (BSF), *Hermetia illucens* (Diptera: Stratiomyidae). Indeed, BSF larvae can be reared on a wide range of decaying organic substrates and, thanks to their high protein and lipid content, they represent a valuable source of macromolecules useful for different applications (e.g., production of feedstuff, bioplastics, and biodiesel), thus contributing to the development of circular economy supply chains for waste valorization. However, decaying substrates bring the larvae into contact with different potential pathogens that can challenge their health status and growth. Although these life strategies have presumably contributed to shape the evolution of a sophisticated and efficient immune system in this dipteran, knowledge about its functional features is still fragmentary. In the present study, we investigated the processes underpinning the immune response to bacteria in *H. illucens* larvae and characterized their reaction times. Our data demonstrate that the cellular and humoral responses in this insect show different kinetics: phagocytosis and encapsulation are rapidly triggered after the immune challenge, while the humoral components intervene later. Moreover, although both Gram-positive and Gram-negative bacteria are completely removed from the insect body within a few hours after injection, Gram-positive bacteria persist in the hemolymph longer than do Gram-negative bacteria. Finally, the activity of two key actors of the humoral response, i.e., lysozyme and phenoloxidase, show unusual dynamics as compared to other insects. This study represents the first detailed characterization of the immune response to bacteria of *H. illucens* larvae, expanding knowledge on the defense mechanisms of this insect among Diptera. This information is a prerequisite to manipulating the larval immune response by nutritional and environmental factors to increase resistance to pathogens and optimize health status during mass rearing.

## Introduction

In recent years, the possibility of rearing saprophagous insects on organic residues has been attracting increasing interest as insect-mediated bioconversion not only represents a strategy to valorize waste, but also to obtain bioproducts, thus generating circular economy value chains (1). In this scenario, the larvae of the black soldier fly (BSF), *Hermetia illucens* (Diptera: Stratiomyidae), play a key role since they have an outstanding ability to grow on a variety of by-products of different supply chains (2–4) and larval proteins can be used not only for formulating feedstuff for poultry, pigs, and fish (5), but also for manufacturing bioplastics (6). Moreover, BSF larvae represent a valuable source of lipids for producing biodiesel (7), chitin (8), and antimicrobial peptides (9).

Great effort has been invested to improve the quality of BSF larvae in terms of protein and lipid content (10–12), mainly by modulating the formulation of their feeding substrate, rearing temperature, humidity, and larval density. These parameters have, however, completely disregarded the fact that most of BSF-based applications involve the use of decaying waste substrates, thus potentially bringing the insect into contact with different pathogens that can challenge its health status and development. Only a few studies have evaluated the ability of *H. illucens* larvae to reduce the presence of pathogens in the rearing substrates (13, 14), although Huang and colleagues (15) have demonstrated that this insect can reduce pathogenic bacteria in the ingested substrate by means of gut immune mechanisms. This finding suggests that BSF larvae have a particularly efficient immune system, ensuring growth and development even in unhealthy organic substrates full of pathogens. However, information on the immune system of BSF larvae is incomplete (16), hindering further exploitation of this insect.

The immune system of insects possesses a sophisticated set of cellular and humoral innate mechanisms that can be rapidly activated in the presence of infections. The main actors of the cellular response are hemocytes, circulating cells involved in phagocytosis, encapsulation, and nodulation of non-self-antigens (17). In parallel, the humoral response is triggered by the interaction of Pattern Recognition Receptors (PRRs), located on the surface of insect cells, and Pathogen-associated Molecular Patterns (PAMPs) such as peptidoglycans, lipopolysaccharides, and β-1,3 glucans that are present on the pathogen surface (18). Humoral responses include the phenoloxidase (PO) system, an enzymatic cascade whose activation culminates with hemolymph clotting and melanin production, antimicrobial peptides (AMPs), lysozyme, and reactive oxygen species (19–21). Although the activation pathways are highly conserved in insects, the triggered cellular and humoral responses can differ between orders and species (18). In some Coleoptera, for example, the maximum number of hemocytes involved in the response to bacteria can be observed just a few hours after infection (22, 23), while in *Anopheles gambiae* this requires 24 hours (24). This finding indicates that, after an immune challenge, different amounts of time are required for circulating hemocytes to proliferate and thus counteract the threat. The pathways that regulate the humoral responses can greatly differ, too. For example, although the honey bee, *Apis millifera*, retains the major pathways typical of insect immune responses, the number of genes involved in the response to different non-self-antigens, including recognition, signaling, and effectors molecules, is reduced by one-third compared to *Drosophila melanogaster* (25), although this difference might be related to the existence of collective immunity that guarantees defenses against diseases in social insects (26). Another example comes from the pea aphid, *Acyrtosiphon pisum*. In this insect, the highly conserved peptidoglycan receptor proteins, as well as other IMD-associated genes involved in the defense against Gram-negative bacteria, including the AMPs Defensin and Cecropin, appear to be missing and Gram-positive bacteria are likely recognized by Gram-negative binding proteins or other receptors (27). Moreover, despite bacterial infections inducing the activation of lysozyme in that aphid, this enzyme does not seem to have a direct role in the host immune response against bacteria (28). Regarding the Diptera, although the immune mechanisms have been exhaustively and comprehensively characterized in the model *D. melanogaster*, it could be informative to investigate the peculiarities of the immune response in those species that have particular lifestyles. In the housefly *Musca domestica*, for example, many immune-related genes significantly expanded and diversified during evolution, as an adaptation to highly septic environments (29).

To our knowledge, and apart from the AMPs, only one study has investigated the larval immune system of BSF (16): here, the authors performed preliminary work on some elements of the humoral response, such as phenoloxidase, lysozyme, and antimicrobial peptides, completely overlooking the cellular mechanisms. More attention has been paid to the AMPs due to biotechnological applications and medical interests, some of which have been recently characterized and cloned from BSF larvae (30–33). Moreover, Vogel et al. (9) performed a RNASeq analysis on whole larvae grown on different rearing substrates (including those with high bacterial loads) and on selected tissues, demonstrating that the AMP expression profile depends not only on the bacterial load of the substrate, but also on its nutrient composition.

In this paper we analyzed the cellular and humoral response of *H. illucens* larvae subjected to bacterial infection, focusing on the dynamics of the reactions. In particular, after injecting different immune elicitors (i.e., Gram-negative and Gram-positive bacteria, and chromatographic beads) into the hemocoel, we investigated the activity of hemocytes in phagocytosis and encapsulation processes, evaluated the antimicrobial activity of hemolymph as such and of the humoral components, analyzed the activity of the PO system and lysozyme, and quantified the expression of genes coding for AMPs.

This study is the first to characterize the immune response of *H. illucens* larvae to bacteria in detail. Resistance to diseases is a key issue for breeding BSF larvae in habitats with high bacterial load, which constantly expose the insect to infections that may reduce its survival and reproductive potential (34). In particular, we analyzed the innate immune response triggered by the Gram-positive saprotrophic bacterium *Micrococcus luteus* and the Gram-negative bacterium *Escherichia coli*, which are widely represented in environmental and soil samples and in organic wastes processed by BSF and other dipteran larvae (35, 36), upon their entrance in the insect hemocoel through lesions on the integument. Understanding the dialogue between cellular and humoral components of the immune system to counteract bacterial infections can be extremely important for maximining the productivity of BSF mass rearing activities and, ultimately, contributing to a wider utilization of the larvae as a viable feed source for livestock and as a sustainable waste management system.

## Materials and Methods

### Insect Rearing

Larvae of *H. illucens* used in this study were obtained from a colony kept for six years at the University of Insubria (Varese, Italy).

Eggs were collected in Petri dishes and maintained at 27 ± 0.5°C until hatching, according to the procedures of Pimentel et al. (37). Neonate larvae were fed with a standard diet for Diptera (50% wheat bran, 30% corn meal, and 20% alfalfa meal mixed at a ratio of 1:1 dry matter:water) (38). Four days after hatching, batches of 300 larvae were transferred to plastic containers (16 x 16 x 9 cm), fed with standard diet, and reared in the dark at 27 ± 0.5°C and 70 ± 0.5% relative humidity. Once at the pupal stage, insects were removed from the substrate and transferred to a cage (70 x 70 x 120 cm) until adult eclosion. Flies were kept at 30 ± 0.5°C, 70 ± 5% relative humidity and with a 12:12 hours light:dark photoperiod, according to Bruno et al. (39).

### Bacterial Strains

The bacterial strains *Escherichia coli* (Strain K12, Sigma-Aldrich, USA) and *Micrococcus luteus* ATCC No. 4698 (Sigma-Aldrich) were used for all the analyses except the encapsulation assay. We selected these bacteria because they are widely represented in organic wastes that can be used by *H. illucens* larvae to grow (36) and can infect the insect through superficial lesions of the integument.

Bacteria were grown in 10 ml of LB broth (Sigma-Aldrich) overnight at 37°C under shaking at 160 rpm. Then, 1-ml aliquots were centrifuged at 1620 x *g* for 15 minutes and cell pellets were washed three times with phosphate buffer (38 mM KH_2_PO_4_, 61.4 mM K_2_HPO_4_, pH 7.4). Finally, after measuring the optical density of the culture at 600 nm (OD_600nm_; one unit of OD_600nm_ corresponds to 4.12 x 10^8^ CFU/ml of *E. coli* and 1.83 x 10^7^ CFU/ml of *M. luteus*), cells were resuspended and diluted in phosphate-buffered saline (PBS, 137 mM NaCl, 2.7 mM KCl, 10 mM Na_2_HPO_4_/KH_2_PO_4_, pH 7.4) to the final concentration for injection into larvae (40).

### Injection of Larvae, Hemolymph Collection, and Control Groups

Last instar larvae were carefully washed with tap water to remove diet debris, then with 0.5% sodium hypochlorite (in tap water, v/v) and 70% ethanol (in distilled water, v/v). To evaluate the activation of cellular and humoral mechanisms by the co-administration of Gram-positive and Gram-negative bacteria, an *E. coli/M. luteus* mix was used to infect the larvae (5µl of the bacterial mix for each larva), unless for those markers where the response of the immune system to Gram-positive or Gram-negative bacteria needed to be specifically evaluated (i.e., antimicrobial activity and phagocytosis). For details on the injection of pHrodo-conjugated bacteria and chromatographic beads, please see the sections below.

All the injections were performed between the third last and penultimate metamere by using a Hamilton 700 10-μl syringe (Hamilton, USA). After injection, larvae were placed in sterile Petri dishes at 27 ± 0.5°C and 70 ± 0.5% relative humidity without food until bleeding.

Hemolymph was collected by piercing the larvae, previously anesthetized on ice, between the last and penultimate metamere with a sterile needle. Each experiment was performed by pooling samples of hemolymph collected from at least 15 larvae and 3 experiments were performed for each test.

As the aim of the present work was to analyze the immune response triggered by the entrance of bacteria through lesions on the body surface, uninjected larvae (naïve) were considered as control groups for all the experiments, as detailed below. To exclude potential side effects of the puncture or the buffer used to resuspend the bacteria, larvae punctured with a sterile needle only or injected with sterile PBS (5 µl) were analyzed at different time points (negative controls) (**Figure S1**). Additional controls were performed by examining starved naïve larvae at different time points (3, 6, 14, 24, and 48 hours) to exclude possible effects of food deprivation on different immune markers (**Figure S2**).

### Determination of the Optimal Bacterial Dose for the Infection of Larvae

The larval immune response was triggered by injecting a mixture of Gram-negative and Gram-positive bacteria into the hemocoel. To determine the adequate bacterial dose for infecting insects, larvae were injected with 5 μl of different concentrations of an *E. coli/M. luteus* mix (10^4^, 10^5^, 10^6^, 10^7^, 10^8^, and 10^9^ CFU/ml) and their mortality was monitored every 24 hours for 3 days. Twenty-five larvae for each condition were used and the experiment was conducted in triplicate. This preliminary experiment was carried out to define the bacterial dose at which the immune system of BSF larvae could be stimulated without causing significant insect mortality during the time considered for the analyses. According to the results obtained in this assay (**Figure S3**), larvae were injected with 5µl of *E. coli/M. luteus* mix at a concentration of 10^5^ CFU/ml for all the experiments, unless otherwise specified.

### Evaluation of Antimicrobial Activity of the Hemolymph

#### CFU Count by the Spread-Plating Method

To determine the bacterial load (CFU/ml) in the hemocoel, hemolymph was collected 6, 14, 24, and 48 hours after infection with *E. coli* or *M. luteus* at a concentration of 10^5^ CFU/ml. Samples were diluted 1:100 with sterile PBS and then plated onto 20 ml of LB broth agar (Sigma-Aldrich). The plates were incubated at 37°C for 24 hours and then colonies were counted. The CFU/ml was calculated as CFU/ml=n° colonies x dilution factor.

#### CFU Count by the Track-Dilution Method

Larvae were injected as described in the section *“Injection of larvae, hemolymph collection, and control groups”* and placed in sterile Petri dishes for 6, 14, 24, or 48 hours. After collection, hemolymph was centrifuged at 250 x *g* to remove the hemocytes. Then, 10 µl of cell-free hemolymph were incubated with 90 µl of 10^6^ CFU/ml *E. coli* or *M. luteus* at 37°C for 3 hours, according to Mastore and Brivio (41). After incubation, samples were serially diluted and then 10 µl of each dilution were dropped onto agar plates. Plates were tilted to allow drops to flow downward and then incubated overnight at 37°C. The colonies (CFU) in each plate were counted and the concentration was calculated as CFU/ml=n° colonies x 10 x dilution factor. *E. coli* or *M. luteus* (10^6^ CFU/ml) without the addition of cell-free hemolymph, and sterile PBS were used as controls. Time point 0 (T_0_) corresponded to cell-free hemolymph derived from naïve larvae incubated for 3 hours with 10^6^ CFU/ml *E. coli* or *M. luteus*.

### Hemocyte Counts

To quantify the hemocytes, hemolymph samples were extracted from the larvae 6, 14, 24, or 48 hours after the infection. Hemocytes were counted by loading diluted hemolymph (10 µl hemolymph added to 90 µl of 0.4% Trypan blue, ThermoFisher, USA) into FAST READ 102 counting chambers (Biosigma S.R.L., Italy) according to the manufacturer’s instructions.

### Phagocytosis Assay

To investigate the phagocytic activity of hemocytes, fluorogenic probes that dramatically increase their fluorescence as pH decreases from neutral to acidic were used. For this purpose, 5 μl of pHrodo™ Red *Staphylococcus aureus* BioParticles Conjugate (0.2 mg/ml) or pHrodo™ Green *E. coli* BioParticles Conjugate (0.2 mg/ml) (Molecular Probes, USA) were injected into the larval hemocoel. Five minutes and 1, 14, and 24 hours later, the hemolymph was extracted from the larvae and 200 µl were loaded on round glass coverslips for 15 minutes in the dark to allow cell adhesion. After discarding the liquid fraction, the hemocytes were fixed with 4% paraformaldehyde in PBS for 5 minutes and then washed twice with sterile PBS. Cells were incubated with DAPI (100 ng/ml in PBS) for nuclear detection and then coverslips were mounted on slides with Citifluor (Citifluor Ltd, United Kingdom) (42). The hemocytes were analyzed by using an Eclipse Ni-U fluorescence microscope (Nikon, Japan) equipped with a DS-SM-L1 digital camera (Nikon, Japan). Phagocytic cells were quantified by analyzing five different images for each independent experiment. The experiments were conducted in triplicate for each time analyzed.

### Encapsulation Assay

B-Agarose, B-Sephadex G-100, CM-Sephadex C-25, and DEAE-Sephadex A-25 beads (Sigma-Aldrich) were used to analyze the encapsulation process. The method was adapted from Mastore and Brivio (43). Briefly, beads were first washed three times with sterile PBS and resuspended in the same buffer, centrifuging at 1620 x *g* for 2 minutes after each wash. Of the final preparation (approximately 5 beads/µl), 5 µl were injected into the larvae. The hemolymph was collected at different time points (2, 4, 14, and 24 hours) and diluted 2:1 (hemolymph/medium) (v/v) with Schneider’s Insect Medium. The encapsulation process was evaluated by using an Olympus IX51 microscope (Olympus, Japan) equipped with an Optika C-P20M camera (Nikon). Encapsulation is an extremely dynamic process due to the different number of hemocytes that adhere to the bead over time, therefore, a qualitative approach was used for this assay. The time at which at least 30% of the beads were at least partially covered by hemocytes was selected as starting point of the process. The experiments were conducted in triplicate for each time point analyzed.

### PO System Activity

To monitor the activity of the PO system after infection, larvae were injected with increasing concentrations of the *E. coli/M. luteus* mix (i.e., 10^5^, 10^7^, 10^8^, or 10^9^ CFU/ml). After 7 minutes, (according to preliminary tests this is the time needed to activate the PO system in *H. illucens* larvae), the hemolymph was collected and centrifuged at 250 x *g* for 5 minutes at 4°C to obtain the humoral (cell-free) fraction. The method used to analyze the PO activity was adapted from Garriga et al. (44). Briefly, 10 μl of cell-free hemolymph were added to 990 µL of 8 mM L-Dopa (L-3-4 dihydroxyphenylalanine) (Sigma-Aldrich) in 10 mM Tris-HCl, pH 7.4. Activation of the PO system was also evaluated by incubating 10 μl of cell-free hemolymph collected from larvae infected with 10^5^ CFU/ml of the *E. coli/M. luteus* mix, with 10 μl of β-glucans from *Saccharomyces cerevisiae* (Zymosan^®^, Sigma-Aldrich) in 980 μl of 8 mM L-Dopa in 10 mM Tris-HCl, pH 7.4.

The formation of dopachrome was evaluated with a V-560 double-beam spectrophotometer (Jasco, USA). Before measurement, samples were incubated at room temperature for 10 minutes and the absorbance was measured at 490 nm every 10 minutes. The increase in absorbance over time (ΔAbs) was determined in the linear range of the curve within 60 minutes. The range in which the absorbance values were registered was 0.1-1 according to Harvey (45).

### Lysozyme Activity

Larvae were infected and placed in sterile Petri dishes for 6, 14, 24, and 48 hours. After collecting the hemolymph, the activity of lysozyme was evaluated according to Garriga et al. (44) with slight modifications. Briefly, N-Phenylthiourea (PTU, Sigma-Aldrich) crystals were added to the samples to prevent activation of the PO system. Samples were centrifuged twice at 250 x *g* for 5 minutes at 4°C, and then at 1600 x *g* for 10 minutes at 4°C, collecting the supernatant. Cell-free hemolymph was diluted 1:10 with sterile PBS. Thereafter, 100 µl of the samples were added to 150 µl of *Micrococcus lysodeikticus* (0.45 mg/ml in 30 mM phosphate buffer, pH 7.2; Sigma-Aldrich), with an optical density of 0.6-0.7, which was used as substrate to measure lysozyme relative activity. *M. lysodeikticus* alone and cell-free hemolymph without addition of the bacterium were used as controls. Absorbance at 450 nm was measured every 30 s for 10 minutes in 96-well plates using a Bio-Rad iMark™ Microplate Absorbance reader (Bio-Rad, USA).

### qRT-PCR Analysis

For qRT-PCR analysis, larvae at 3, 6, 14, 24, and 48 hours after the infection were anesthetized on ice and then dissected. The fat body was isolated, frozen in liquid nitrogen, and stored at -80°C until use. In parallel, the hemolymph collected from larvae infected for 3, 6, 14, and 24 hours was isolated as described in the section *“Injection of larvae and hemolymph collection”* and placed in sterile tissue culture Petri dishes (5.5 x 1.5 cm) for 20 minutes in the dark to allow cell adhesion. Afterwards, the hemocytes were washed twice with Schneider’s Insect Medium to remove fat body debris, then scraped from the Petri dish with Trizol Reagent (Life Technologies, USA), and stored at -80°C until use.

RNA was extracted from 30-40 mg of fat body and 10^6^ hemocytes/ml with Trizol Reagent. Samples were treated with TURBO DNA-free Kit (Life Technologies) to avoid genomic DNA contamination and RNA quality was verified through gel electrophoresis.

Retrotranscription was performed with M-MLV reverse transcriptase (Life Technologies). Primers used for qRT-PCR (**Table 1**) were designed based on the sequences of Defensin, Diptericin, Lysozyme, and Ribosomal Protein L5 (RPL5) obtained from *de novo* transcriptome of *H. illucens* larvae (Transcriptome accession number: ERP122672) (46). RPL5 was used as housekeeping gene (34, 39). Real-Time PCR was performed using the iTaq Universal SYBR Green Supermix (Bio-Rad) and the CFX Connect Real-Time PCR Detection System (Bio-Rad), according to (47). Relative expression of the genes was calculated with the 2^-ΔΔCt^ method. Each value was the result of the analysis of five series of samples.

**Table 1:** Sequence of primers used in this study.

Gene name	Transcriptome Accession number	Contig number	Primer sequences
***HiDefensin***	ERP122672	TRINITY_DN10226_C0_G1_I2	F: GCGTTCTATTCTCGTCTTGG
			R: TGCTGTTCCACTACCTGACT
***HiDiptericin***	ERP122672	TRINITY_DN6246_C0_GI_I1	F: CCCAGTGAGCGATGAGGAA
			R: GTGAAGGGTATTGCGTCCAT
***HiLysozyme***	ERP122672	TRINITY_DN12175_C0_G2_I1	F: GCCCAAGGCAAGGTTTACA
			R: TGGCGAGGGTGGTTAGATTC
***HiRPL5***	ERP122672	TRINITY_DN8551_C0_G1_I2	F: AGTCAGTCTTTCCCTCACGA
			R: GCGTCAACTCGGATGCTA

### Statistical Analysis

Statistical analyses were performed using GraphPad Prism version 7.00 (GraphPad software, La Jolla, USA). The antimicrobial and lysozyme activity, AMPs and lysozyme expression, and the activation of PO system were analyzed using One-Way ANOVA followed by Tukey’s multiple-comparison *post hoc* test. Hemocyte counts were analyzed using the Unpaired Student’s *t*-test while survival curves of the larvae were compared using Kaplan-Meier followed by log-rank analysis. All the data were subjected to logarithmic (log_10_) transformation for normality before statistical analysis. Statistical differences between groups were considered significant at a *p*-value < 0.05.

## Results

### Determination of the Optimal Bacterial Dose for the Infection of Larvae

The bacterial concentration used in this study was selected by monitoring larvae injected with different concentrations of the *E. coli*/*M. luteus* mix (from 10^4^ to 10^9^ CFU/ml) for up to 72 hours. As shown in **Figure S3**, survival of the larvae was inversely proportional to the bacterial dose administered. In particular, while 100% of the larvae injected with 10^4^ CFU/ml bacteria were alive 72 hours after the infection, higher bacterial doses (10^5^, 10^6^, 10^7^, and 10^8^ CFU/ml) progressively decreased the number of live larvae over time (72%, 64%, 60%, and 16%, respectively, at 72 hours). All the insects died within 24 hours at the highest concentration of bacteria (10^9^ CFU/ml). According to these results, for all the subsequent experiments, larvae were infected by injecting 10^5^ CFU/ml of the bacterial mix as this was the lowest concentration able to reduce the welfare of the larvae, without causing high mortality, thus allowing us to monitor the immune response over time.

### Analysis of the Antimicrobial Activity in the Hemolymph

**Figure 1:**
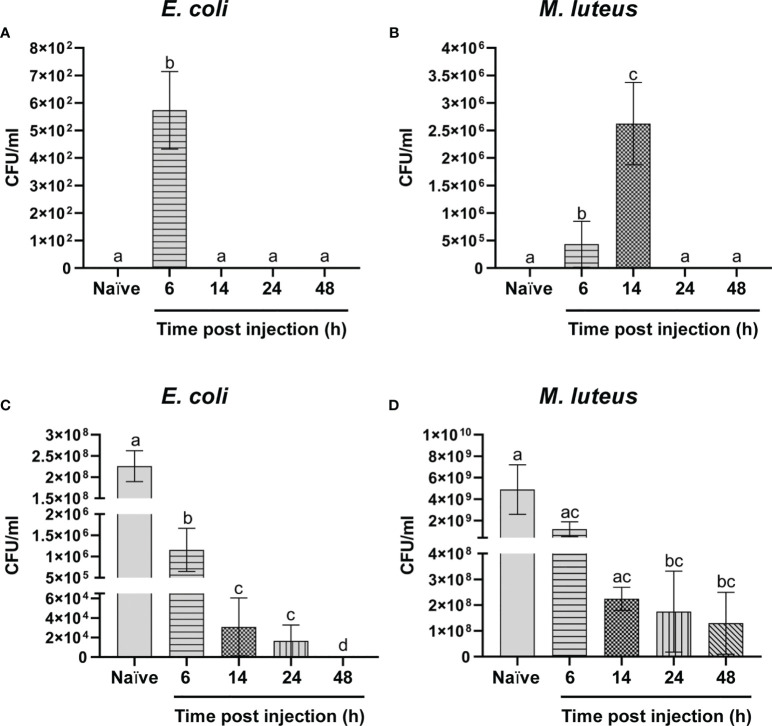
Analysis of the antimicrobial activity. **(A, B)** Humoral and cellular activity against *Escherichia coli* **(A)** and *Micrococcus luteus* **(B)**. **(C, D)** Humoral antimicrobial activity against *E. coli* **(C)** and *M. luteus* **(D)**. Values represent mean ± s.e.m. Different letters indicate statistically significant differences among treatments (One-Way ANOVA: **A**) *F*_4-10_ = 91.65, *p* < 0.0001; **B**) *F*_4-10_ = 74.62, *p* < 0.0001; **C**) *F*_4-10_ = 52.36, *p* < 0.0001; **D**) *F*_4-10_ = 4.694, *p* < 0.0216).

The bacterial load in the hemolymph was evaluated at different time points after the bacterial infection. While the *E. coli* concentration dropped to zero 14 hours after the infection (**Figure 1A**), *M. luteus* proliferation at 6 hours (4.34 ± 4.2 x 10^5^ CFU/ml) was higher than that observed for *E. coli* at the same time point after infection (5.73 ± 1.4 x 10^2^ CFU/ml) and even increased at 14 hours (2.62 ± 0.8 x 10^6^ CFU/ml). However, no CFUs were detected 24 hours from the infection onwards for the two bacteria (**Figures 1A, B**).

The results of a track dilution method showed a time-dependent trend in the antimicrobial activity of the cell-free hemolymph against *E. coli*. In fact, the initial bacterial concentration (2.26 ± 0.4 x 10^8^ CFU/ml) dropped to 1.16 ± 0.5 x 10^6^ CFU/ml and to 3.08 ± 2.9 x 10^4^ CFU/ml by adding humoral components of cell-free hemolymph collected from larvae at 6 and 14 hours after the infection, respectively, and no colonies were found by testing hemolymph from larvae at 48 hours after the infection (**Figure 1C**). The antimicrobial activity against *M. luteus* showed a different trend. Although the initial concentration of this bacterium (4.9 ± 2.3 x 10^9^ CFU/ml) constantly fell at different time points, a significant number of colonies were still observed in hemolymph collected at 48 hours (**Figure 1D**). These data demonstrate that bacterial infection is rapidly reduced due to the cooperation of cellular and humoral responses (**Figures 1A, B**) and indicate that the cellular response plays a key role in counteracting the infection of *M. luteus* since, unlike the response to *E. coli*, cell-free hemolymph collected from larvae at 48 hours after infection is unable to completely inhibit the bacterial growth (**Figures 1C, D**).

### Cellular Response

#### Hemocyte Number

**Figure 2:**
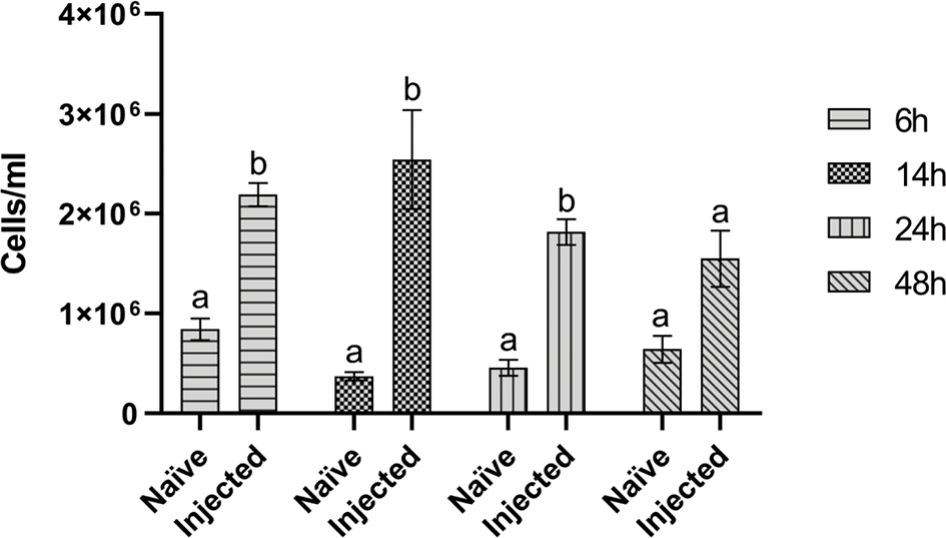
Hemocyte counts. Hemocyte counts of naïve larvae (Naïve) and larvae infected with 10^5^ CFU/ml *E. coli/M. luteus* mix (Injected) at different time points (6, 14, 24, and 48 hours after infection). Values represent mean ± s.e.m. Different letters indicate statistically significant differences among naïve and injected larvae (Unpaired Student’s *t*-test: for 6h *t* = 3.289, *df* = 5, *p* = 0.0217; for 14h *t* = 7.864, *df* = 5, *p* = 0.0005; for 24h *t* = 4.661, *df* = 4, *p* = 0.0096; for 48h *t* = 1.595, *df* = 4, *p* = 0.1859).

To evaluate possible variations in the number of circulating hemocytes after infection with the *E. coli/M. luteus* mix, hemocytes were counted. A significant difference between the hemocyte number in control (8.45 ± 2.2 x 10^5^ cells/ml) and infected (2.19 ± 0.2 x 10^6^ cells/ml) larvae 6 hours after injecting the bacteria was observed (**Figure 2**). This difference remained stable up to 24 hours (4.59 ± 1.4 x 10^5^ cells/ml vs 1.82 ± 0.2 x 10^6^ cells/ml) and disappeared 48 hours after infection (6.43 ± 2.3 x 10^5^ cells/ml vs 1.55 ± 0.5 x 10^6^ cells/ml) (**Figure 2**).

#### Phagocytosis

**Figure 3:**
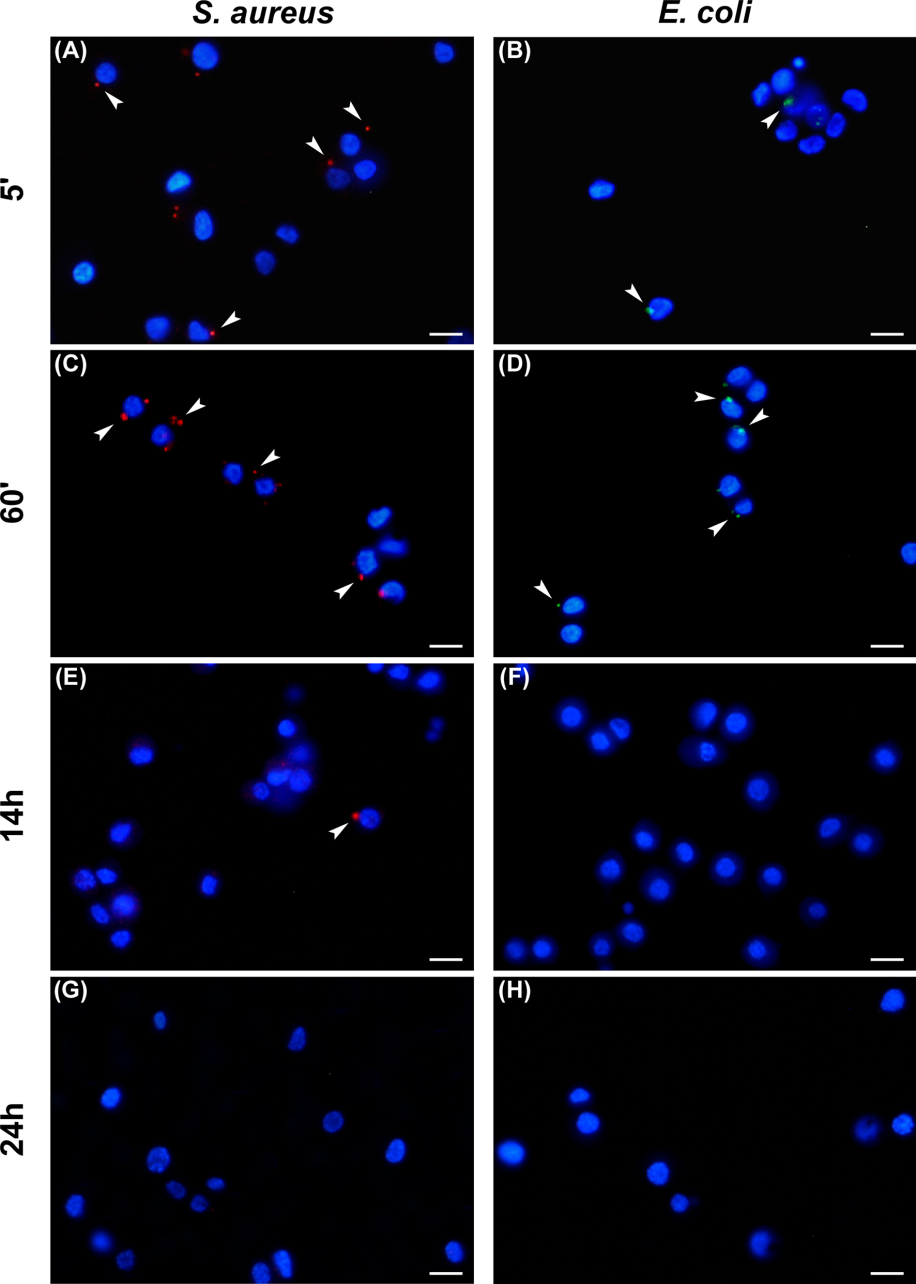
Analysis of phagocytosis. Hemocytes undergoing phagocytosis 5 minutes **(A, B)**, 60 minutes **(C, D)**, 14 hours **(E, F)**, and 24 hours **(G, H)** after injection of *Staphylococcus aureus* **(A, C, E, G)** and *Escherichia coli* **(B, D, F, H)** conjugated with pHrodo. Arrows indicate phagocytosed bacteria. Bars: 10 µm.

To analyze the trend of the phagocytosis process over time, Gram-positive and Gram-negative bacteria conjugated with pHrodo fluorophores were injected in the larvae and the hemocytes examined at different time points (**Table 2**). For both bacterial species, a time-dependent increase in phagocytic activity was observed up to 1 hour after injection in the larvae. In particular, while 5 minutes after infection the counts for hemocytes that had engulfed fluorescent bacteria were 22 ± 4% and 45 ± 3% for *E. coli* and *S. aureus*, respectively (**Figures 3A, B**), an increasing number of active phagocytes was observed after 1 hour (41 ± 2% and 86 ± 6% for *E. coli* and *S. aureus*, respectively) (**Figures 3C, D**). Moreover, a few, engulfed Gram-positive bacteria were detected after 14 hours (6 ± 1%) (**Figures 3E, F**), while no signal was found at 24 hours for either bacterial species (**Figures 3G, H**).

**Table 2:** Quantification of cells undergoing phagocytosis.

Time post infection	Percentage of phagocytic cells	
	*E. coli*	*S. aureus*
**5 minutes**	22 ± 4^a^	45 ± 3^a^
**60 minutes**	41 ± 2^b^	86 ± 6^b^
**14 hours**	0 ± 0^c^	6 ± 1^c^
**24 hours**	0 ± 0^c^	0 ± 0^d^

Values represent mean ± s.e.m. Different letters indicate statistically significant differences among cells undergoing phagocytosis (E. coli or S. aureus) and non-phagocytic cells at different time points (One-Way ANOVA: for E. coli F_3-11_ = 876.8, p < 0.0001; for S. aureus F_3-11_ = 910.8, p < 0.0001).

#### Encapsulation

**Figure 4:**
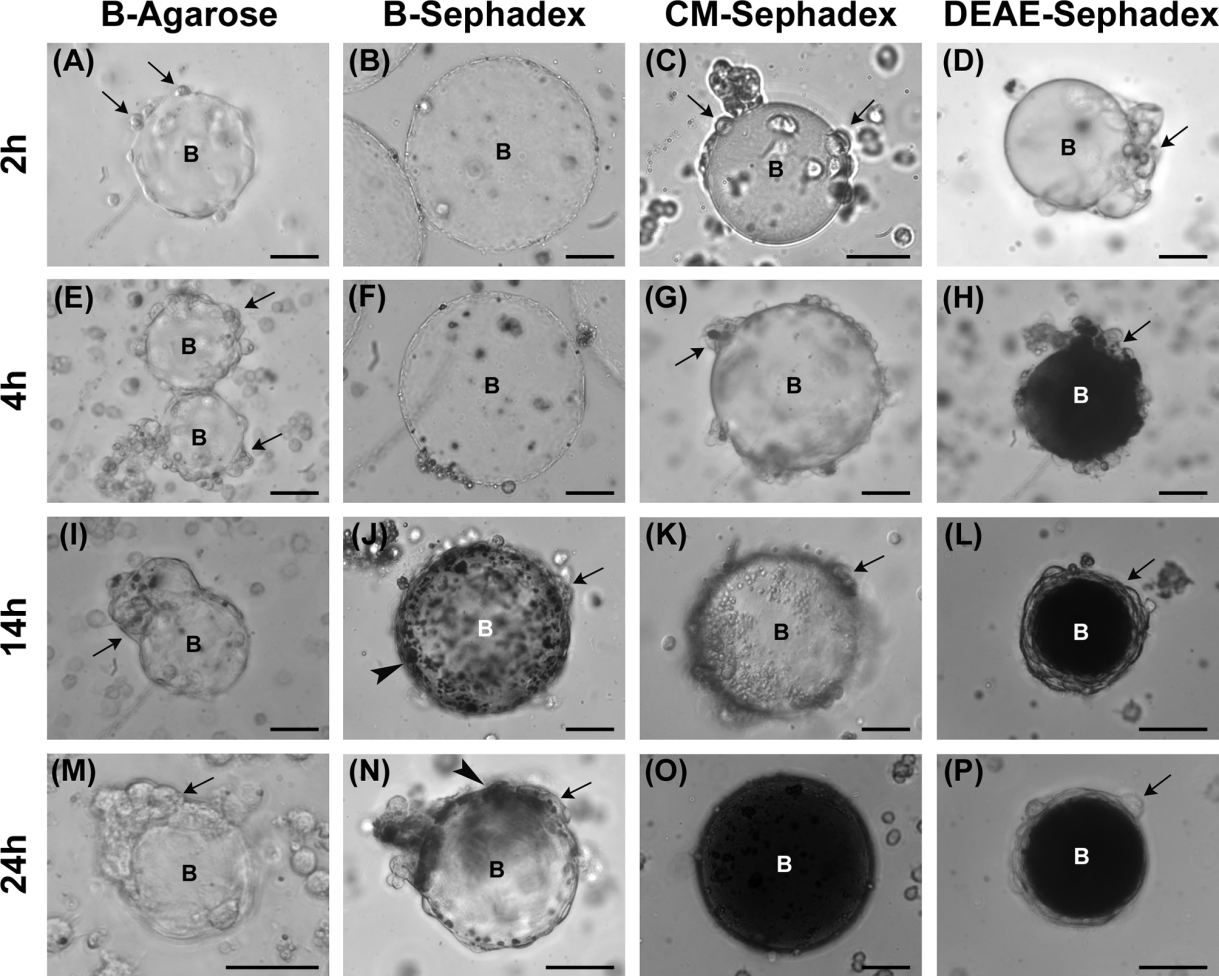
Analysis of encapsulation. Effect of B-Agarose **(A, E, I, M)**, B-Sephadex **(B, F, J, N)**, CM-Sephadex **(C, G, K, O)**, and DEAE-Sephadex **(D, H, L, P)** beads on encapsulation and melanization. Hemolymph was analyzed 2 **(A–D)**, 4 **(E–H)**, 14 **(I–L)**, and 24 (M-P) hours after injecting the beads in the larvae. Arrows, adherent hemocytes; arrowheads, melanin deposition; B, beads. Bars: 40 µm.

We analyzed the qualitative trend of the encapsulation process and melanin deposition over time by injecting different beads into the larval hemocoel. First, we compared the effects of two types of neutrally charged beads, i.e., agarose and dextran. B-Agarose beads partially surrounded by hemocytes were visible 2 hours after being injected in the larva (**Figure 4A**) and, despite the extensive recruitment of cells adhering to the beads over time (**Figures 4E, I, M**), production and deposition of melanin were never observed. Conversely, beads surrounded by hemocytes and melanin deposition could be observed from 14 hours onwards in larvae injected with B-Sephadex beads (**Figures 4B, F, J, N**).

We then investigated whether the charge of the beads could affect the encapsulation process and melanin deposition. For this, negatively and positively charged dextran beads were tested. In larvae treated with CM-Sephadex beads (negatively charged), hemocytes began to adhere to the beads very quickly (2-4 hours after the injection) (**Figures 4C, G**). Although the beads were not completely surrounded by hemocytes at longer times after injection (**Figure 4K**), full melanization could be seen 24 hours after being injected (**Figure 4O**). Conversely, for DEAE-Sephadex beads (positively charged) (**Figure 4D**), close adhesion of the cells to the foreign body and its melanization could already be observed 4 hours after their injection (**Figure 4H**), and a typical melanotic capsule could be detected after 14 hours (**Figures 4L, P**).

### Humoral Response

#### PO System

**Figure 5:**
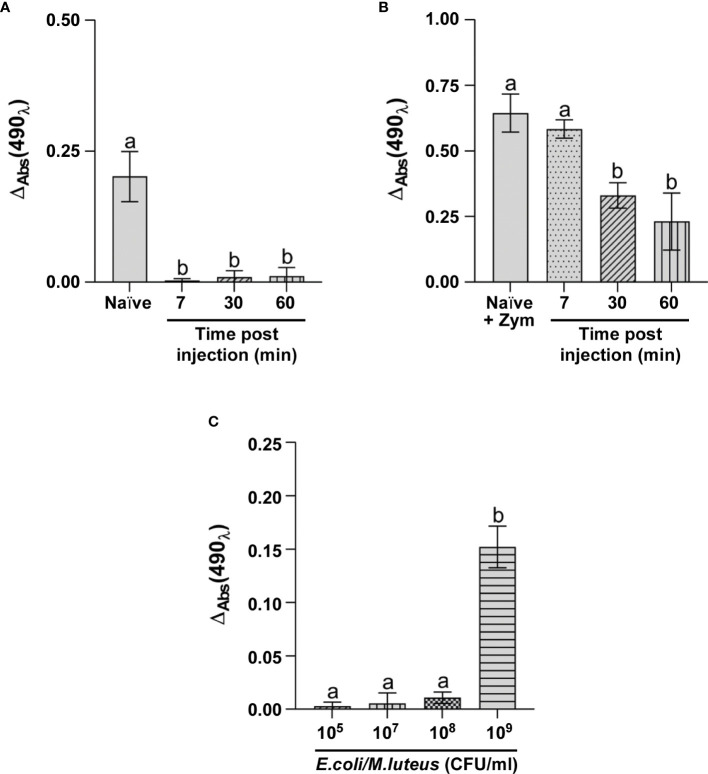
Analysis of PO system activation. ΔAbs at 490 nm of phenoloxidase in **(A)** hemolymph of naïve larvae (Naïve) and larvae infected for 7, 30 and 60 minutes with 10^5^ CFU/ml of *E. coli*/*M. luteus* mix; **(B)** hemolymph isolated from naïve larvae (Naïve + Zym) and larvae infected for 7, 30, and 60 minutes with 10^5^ CFU/ml of *E. coli*/*M. luteus*, treated with Zymosan; **(C)** hemolymph isolated from larvae infected for 7 minutes with different concentrations of *E. coli*/*M. luteus* mix. Values represent mean ± s.e.m. Different letters indicate statistically significant differences among treatments (One Way ANOVA: **A**) *F*_3-8_ = 41.54, *p* < 0.0001; **B**) *F*_3-8_ = 22.95, *p* = 0.0003; **C**) *F*_3-8_ = 122.7, *p* < 0.0001).

Activation of the PO system was first evaluated in larvae injected with 10^5^ CFU/ml *E. coli*/*M. luteus* mix. The ΔAbs value in the cell-free hemolymph from control larvae was significantly higher than that of larvae infected for 7 minutes (**Figure 5A**). The pattern was unchanged in hemolymph collected from larvae infected with the bacterial mix for longer times (30 and 60 minutes) (**Figure 5A**), suggesting the possibility of a potential inhibition/inactivation of the PO system.

Zymosan, a specific activator of the PO system was added to the cell-free fraction of hemolymph from infected larvae to verify enzymatic system functioning (**Figure 5B**). The results showed a consistent activation of phenoloxidase under this condition and values were comparable to those of hemolymph from naïve larvae treated with Zymosan (**Figure 5B**). Moreover, although the addition of Zymosan to the hemolymph collected from larvae infected for longer times (30 and 60 minutes) reduced PO system activity compared to larvae infected for 7 minutes (**Figure 5B**), Zymosan always activated the enzyme system.

As these experiments with Zymosan demonstrated PO system functioning, we evaluated whether activation might possibly depend on the bacterial concentration. Therefore, we compared phenoloxidase activation in the cell-free fraction of hemolymph from larvae injected with increasing concentrations of the bacterial mix (10^5^, 10^7^, 10^8^, and 10^9^ CFU/ml) (**Figure 5C**). The data showed that the PO system is activated at a concentration of bacteria higher than or equal to 10^9^ CFU/ml, indicating that this concentration is the threshold for full activation of the enzyme complex.

#### Expression and Activity of Lysozyme

**Figure 6:**
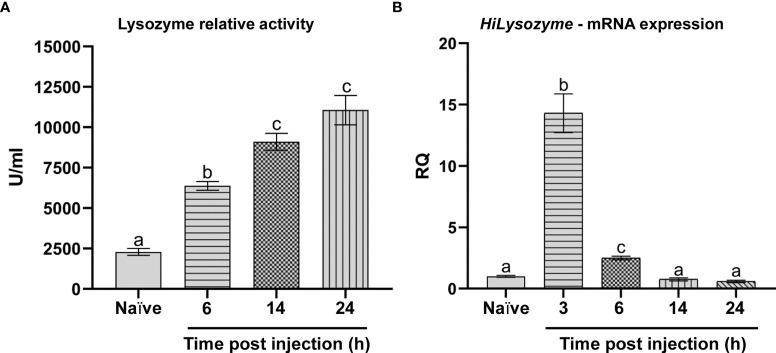
Lysozyme activity and *HiLysozyme* expression. **(A)** Lysozyme relative activity in the hemolymph of naïve and infected larvae at different time points. **(B)** *HiLysozyme* mRNA levels in hemocytes of naïve and infected larvae at different time points. Naïve larvae starved for 6 and 3 hours were used as controls for the activity and the expression of lysozyme, respectively. Values represent mean ± s.e.m. Different letters indicate statistically significant differences among treatments (One-Way ANOVA: **A**) *F*_4-10_ = 128.1, *p* < 0.0001; **B**) *F*_4-10_ = 107.2, *p* < 0.0001).

In cell-free hemolymph from naïve larvae, we measured a basal activity of lysozyme (2288 ± 211 U/ml), which progressively increased after infection with the bacterial mix (**Figure 6A**). Specifically, the activity of the enzyme increased from 6633 ± 133 U/ml after 6 hours up to 9100 ± 900 U/ml after 14 hours and 11316 ± 687 U/ml at 24 hours (**Figure 6A**).

We also analyzed the transcription of *HiLysozyme* both in the fat body and in hemocytes. While the mRNA levels of this gene in the fat body of infected larvae were not significantly higher than in controls (data not shown), levels in hemocytes varied over time. In fact, a rapid and marked increase (15-fold) in *HiLysozyme* mRNA levels was observed within 3 hours after infection. Then, mRNA levels decreased at 6 hours and returned to basal values after 14 hours (**Figure 6B**).

#### Expression of Antimicrobial Peptides

**Figure 7:**
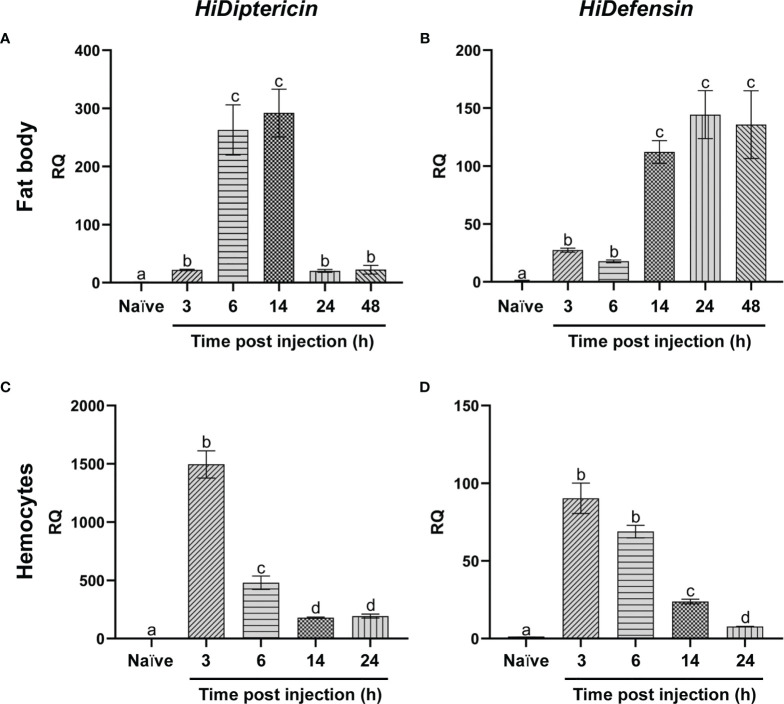
qRT-PCR analysis of antimicrobial peptides. **(A, C)** mRNA levels of *HiDiptericin* in the fat body **(A)** and hemocytes **(C)** of naïve and infected larvae at different time points; **(B, D)** mRNA levels of *HiDefensin* in the fat body **(B)** and hemocytes **(D)** of naïve and infected larvae at different time points. Naïve larvae starved for 3 hours were used as controls. Values represent mean ± s.e.m. Different letters indicate statistically significant differences among treatments (One-Way ANOVA: **A**) *F*_5-12_ = 178.8, *p* < 0.0001; **B**) *F*_5-12_ = 127, *p* < 0.0001; **C**) *F*_4-10_ = 532.6, *p* < 0.0001; **D**) *F*_4-10_ = 658.3, *p* < 0.0001).

The expression of *HiDiptericin* and *HiDefensin* was investigated both in the fat body and in hemocytes. These two representative AMPs were selected to obtain information about AMP response to Gram-negative and Gram-positive bacteria, respectively. In the fat body the slight increase in mRNA levels of both AMPs 3 hours after the infection changed at later times. In particular, a >250-fold increase in the expression of Diptericin was observed at 6-14 hours; then, mRNA levels markedly decreased within 24 hours (**Figure 7A**). Conversely, Defensin expression increased later compared to Diptericin (110-fold change at 14 hours) and levels remained stable up to 48 hours (**Figure 7B**).

The expression levels of the two AMPs in hemocytes displayed a peak within 3 hours from the infection [1400-fold for Diptericin and 90-fold for Defensin (**Figures 7C, D**)]; then, mRNA levels gradually decreased at the later times (**Figures 7C, D**).

## Discussion

**Figure 8:**
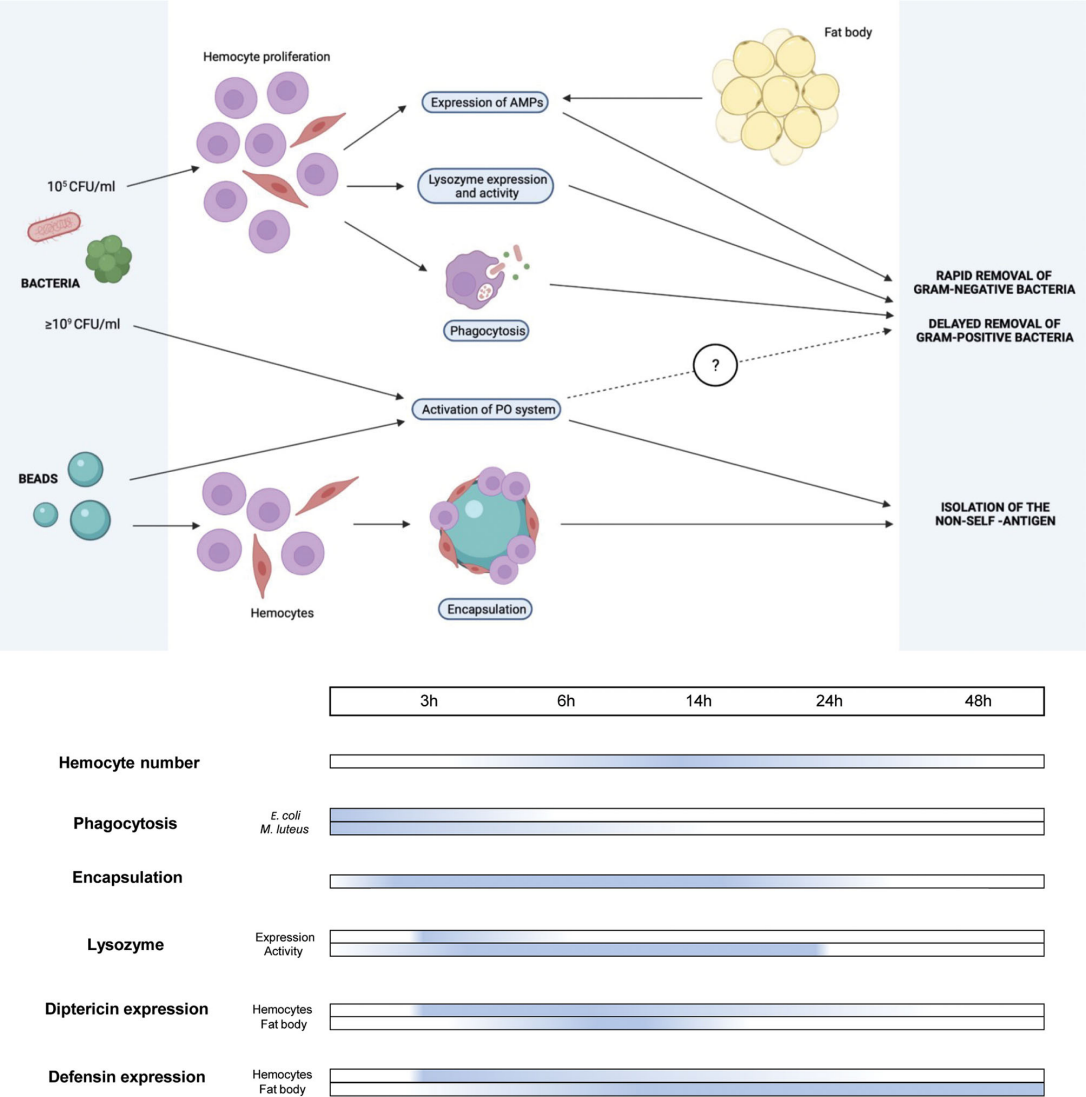
Schematic representation of cellular and humoral responses in *H. illucens* larvae and activation timing.

Over the last few years, considerable effort has been devoted to investigating BSF biology. In particular, the literature provides information regarding the functional properties of its digestive (39, 46, 48–51) and reproductive (52, 53) system, the fat body (37), and other organs and structures (54–58). Although interest in these aspects is increasing, information about the immune system of BSF is still fragmentary. In fact, different studies have focused attention on the antimicrobial activity of BSF larval extracts (9, 31, 32, 59) while, to the best of our knowledge, only one considered the response of the immune components after an immune challenge (16). However, it must be highlighted that, in contrast to this latter study in which larvae were infected by pricking with an unknown bacterial load, the present study applied a controlled immune challenge by injecting a defined amount of bacteria into the hemocoel. The bacterial load for the infections was accurately selected to reduce insect welfare, stimulating the immune system, but ensuring a high survival rate of the larvae to analyze the cellular and humoral responses in detail over time (**Figure 8**).

In insects, a continuous cross-talk between hemocytes and humoral molecules is fundamental for maintaining the hemolymph devoid of pathogens and parasites (20, 60). During infection, the cellular response is triggered when transmembrane or soluble PRPs recognize PAMPs and usually ensues more rapidly than the humoral response to quickly reduce the pathogen load in the hemolymph. The few remaining microorganisms not eliminated by hemocytes are killed by humoral molecules (61). Our results confirm this sequence of events and demonstrate the need for synergic action of the two components of the immune system to efficiently remove invading bacteria in *H. illucens* larvae. In particular, the antimicrobial activity against *E. coli* and *M. luteus* elicited by the whole hemolymph and that of the cell-free fraction were significantly different, with a quite rapid clearance of bacteria displayed by the former versus the inability of the latter to kill both bacteria in a short time. Moreover, unlike *E. coli*, which is totally removed within 48 hours by both the hemolymph and cell-free fraction, the latter was unable to eliminate *M. luteus* even at longer times from the infection. The longer time required for humoral molecules to remove *M. luteus* could be due to the potential capability of this bacterium to elude the action of AMPs, as previously demonstrated for some Gram-positive bacteria (62–64). In addition, the amount of time needed to fully activate the Toll and IMD pathways, which are involved in the defense against Gram-positive and Gram-negative bacteria, respectively, could be different, thus causing a quicker elimination of *E. coli* than of *M. luteus* (65). These two hypotheses are part of a more general strategy that leads the immune system of insects to selectively activate specific responses to minimize the cost of the immune defense (66). Different reaction times against specific foreign agents can thus be the result of this cost minimization criterion, which, in turn, can sometimes reduce the efficacy of the immune response. The lack of a bactericidal action of cell-free hemolymph against *M. luteus* and the need for hemocytes to support the immune response and counteract this pathogen is confirmed by the marked increase in circulating cells, starting 6 hours after the infection, which is maintained for longer times.

Although hemocytes are responsible for producing humoral molecules (67, 68), they are mainly involved in phagocytosis, encapsulation, and nodulation (17). In Diptera, phagocytosis is triggered very rapidly after an immune challenge [from 5 to 15 minutes from the infection depending on the type of bacteria (69–72)] and can last up to 24 hours (70, 71). Here, we showed that phagocytosis is rapidly activated by both Gram-positive and Gram-negative bacteria, also in *H. illucens* larvae, as confirmed by pHrodo-conjugated bacteria, which are phagocytosed by circulating hemocytes only 5 minutes after the infection. In contrast to other Diptera such as *Armigeres subalbatus* and *Aedes aegypti* (70, 71), the engulfment activity in *H. illucens* larvae declines rapidly and is absent at the longer time points (i.e., 24 hours). This difference could be attributed to the infection protocol as, here, we injected a defined and limited concentration of Gram-positive and Gram-negative bacteria in the larvae instead of using an “uncontrolled” pricking infection (70, 71). However, a more intriguing hypothesis could be the existence in BSF of phagocytes that are quicker and more efficient in removing bacteria than in other Diptera as a possible adaptation of these larvae to substrates with high bacterial loads, which require a very rapid and robust cellular immune response to counteract potential, dangerous infections.

We showed that the kinetics of encapsulation and melanin deposition in BSF are affected by the non-self-antigen. In fact, as in other Diptera and Lepidoptera (73, 74), melanotic capsule formation strongly depends on the matrix, with agarose beads that are never melanized although recognized and surrounded by hemocytes, in contrast to dextran beads, which are rapidly encapsulated and melanized. Moreover, a correlation between the bead charge and the degree of melanin deposition has been observed. In particular, positive-charged beads elicited the strongest response in terms of melanin deposition, which was also observed in other Diptera (75) and Lepidoptera (74, 76). As encapsulation in insects usually involves the joint action of different hemocyte populations (i.e., granulocytes, involved in the release of lectins for PO activation, actively recruit plasmatocytes, which leads to capsule formation) (77, 78), ultrastructural analyses of the encapsulated beads are mandatory to address how the three hemocyte populations reported in *H. illucens* larvae (16) take part in this process. Moreover, our results may indicate how BSF larvae react to larger parasites characterized by different surface charge (79).

The lack of activation of the PO system at low bacterial concentrations indicates that melanization depends on the bacterial load in the hemocoel and here we have demonstrated the existence of a threshold level over which melanin is produced. The inhibition of melanin formation has been observed in immunized *Galleria mellonella* larvae (80), too, and it could be mediated by the presence in the hemolymph of active inhibitors of the enzymatic cascade, such as serpins and CLSP2 protein (81, 82). These inhibitors, which are only triggered when the PO cascade is activated, keep melanin formation under strict control, preventing an unnecessary production/overproduction of this pigment, which can be highly toxic for the insect itself (83–85). Experiments with hemolymph from naïve larvae indicate that such regulatory mechanisms exist in *H. illucens*. In fact, these inhibitors are present in their inactive form since the PO system has not been activated yet: once isolated from the larval body, simple exposure to the air activates the PO system, which is not subjected to such negative regulation, as previously seen in *Calliphora* larvae (86). Therefore, when *H. illucens* larvae experience a limited bacterial infection, phagocytosis, antimicrobial peptide production, and lysozyme activity are sufficient to counteract the threat, inhibiting at the same time the activation of the PO system. Conversely, in the presence of a significant bacterial load in the hemocoel, these cellular and humoral systems are unable to efficiently contain the bacterial spread and the PO system is activated, leading to melanin production and release in the larval body (**Figure 8**). In BSF an additional inhibitory effect on this enzyme system could be exerted by lysozyme and AMPs, two known negative regulators of PO (87, 88), as they increase in parallel with inhibition of the PO system.

Interestingly, expression levels of *HiLysozyme* in the fat body did not increase after injecting bacteria, suggesting that the constitutive activity of this enzyme can counteract the bacterial threat, at least at the beginning of the infection (89). Moreover, the initial degradation of bacteria mediated by the constitutive activity of lysozyme could be useful for generating PAMPs, which stimulate the host immune system to obtain a stronger response against non-self-antigens (90). A temporally limited increase in mRNA levels was registered in hemocytes, indicating that circulating cells are the main source of lysozyme production during infection (91, 92) and their synthesizing activity is needed to maintain high levels of lysozyme in the hemolymph. Accordingly, the activity of this enzyme also remained elevated at longer times after the infection (24 hours), although no more bacterial colonies were detected. This pattern could be useful to the larva to promptly respond to a second infection, suggesting the existence of immune priming in BSF (93).

In contrast to lysozyme, the transcription of both AMPs was highly stimulated by infection both in the fat body and in hemocytes. In particular, the early increase in AMP mRNA levels in circulating cells (3-6 hours), probably required to start challenging the infection, is rapidly supported by a later, but prolonged (6-48 hours) production of AMPs in the fat body, which represents the main organ involved in their synthesis (94). Interestingly, while the transcription of Diptericin, which is active only against Gram-negative bacteria (95), returns to low levels 24 hours after the infection, the trend observed for Defensin, which is active against Gram-positive bacteria (96), is different. In fact, although no colonies of *M. luteus* are found 24 hours after the infection, the expression levels of Defensin remain high up to 48 hours. This pattern may be due to the enhanced ability of Gram-positive bacteria to counteract the immune response of *H. illucens*; thus, high levels of this AMP are maintained to better counteract a potential, second infection.

Insect immunity with its cellular and humoral arms has evolved under substantial selective pressure to protect the host against foreign invaders (17, 97). The biological significance of the present study lies in its more detailed appreciation of how the speed and kinetics of immune activation influence the ability of the host (i.e., BSF larvae, whose immune system is poorly studied despite the great interest in this insect for its bioconversion ability) to counteract a specific category of invaders (i.e., Gram-positive and Gram-negative bacteria). The data reported herein highlight that the two branches of the immune system have different activation dynamics in this insect as phagocytosis and encapsulation are rapidly triggered once the larva is exposed to the foreign antigen, while the humoral components intervene later. This evidence - although important because it fills a gap in our knowledge of BSF larvae - is common among insects, that are able to activate different defense mechanisms against infections *via* different speeds, specificities, and routes (17, 97). The possible variation in numbers and populations of circulating hemocytes, and the rate of their recruitment to overcome the intruders likely affect the extent and time course of phagocytosis and encapsulation among different insects and within the same species depending on the foreign invader (17). Similarly, the variation in the evoking potency of the bacterial cell wall PAMPs, e.g., peptidoglycan or lipopolysaccharides of Gram-positive and Gram-negative bacteria, respectively, and the efficiency of the subsequent response mechanisms may contribute, in part, to the speed and specificity of immune phenomena in different challenged insects, e.g., Orthoptera (98), Lepidoptera (99), Hymenoptera (100), Coleoptera (101) and other Diptera (102). Moreover, the infection route itself can also affect the timing and expression of AMPs. Here, we employed a method to directly deliver pathogens to the larva *via* injection, but it will be necessary to investigate the effects of oral administration of bacteria as well. In fact, injection of the sand fly *Lutzomyia longipalpis* with *M. luteus* or *Serratia marcescens* did not result in increased Defensin expression until 72 hours later (103). Conversely, oral infection strongly induced an acute antimicrobial response by upregulating Defensin expression in both larval and adult stages. Thus, viewed through the lens of evolution, the immune system of modern insects may be so exquisite and fine-tuned to the point of providing apparently paradoxical and contradictory observations.

Summarizing, this study: i) provides new information on the BSF immune system and expands knowledge on the defense mechanisms of Diptera, ii) represents a platform of knowledge for future studies on the immune response of BSF larvae challenged with other infectious agents, such as fungi or viruses, and iii) is a prerequisite to manipulating the larval immune response by nutritional (i.e., the diet) (9) or environmental (i.e., rearing temperature or larval density) (104, 105) factors, to increase resistance to pathogens and optimize health status during mass rearing.

## Data Availability Statement

The raw data supporting the conclusions of this article will be made available by the authors, without undue reservation.

## Author Contributions

DB, MC, and GT designed the study. DB, AuM, MM, and AG performed the research. DB, AuM, MB, MC, and GT analyzed the data. DB, AuM, MFB, AmM, LT, MC, and GT wrote the paper. All authors contributed to the article and approved the submitted version.

## Funding

This work was supported by Fondazione Cariplo (grant number 2020-0900).

## Conflict of Interest

The authors declare that the research was conducted in the absence of any commercial or financial relationships that could be construed as a potential conflict of interest.

The handling editor IE has declared a past co-authorship with one of the authors (GT) at the time of review.

## Publisher’s Note

All claims expressed in this article are solely those of the authors and do not necessarily represent those of their affiliated organizations, or those of the publisher, the editors and the reviewers. Any product that may be evaluated in this article, or claim that may be made by its manufacturer, is not guaranteed or endorsed by the publisher.
