# Supplementary material for: Insights Into the Immune Response of the Black Soldier Fly Larvae to Bacteria
Source: Front Immunol. 2021 Nov 18;12:745160. doi: 10.3389/fimmu.2021.745160 (PMC8636706; doi:10.3389/fimmu.2021.745160)

Supplementary Material

**Supplementary Figure 1 - Effects of needle puncture and PBS injection on the immune response of BSF larvae.**

Control experiments were performed to assess possible effects of puncturing the larva with a sterile needle (Needle) or injecting sterile PBS (PBS) in the hemocoel. Hemocyte number (A), lysozyme activity (B), *HiLysozyme* expression (C), *HiDiptericin* (D) and *HiDefensin* (E) expression in the fat body, *HiDiptericin* (F) and *HiDefensin* (G) expression in hemocytes under these conditions were compared with naïve larvae (Naïve) and larvae infected with 10^5^ CFU/mL of *E. coli/M. luteus* mix (Injected). Values represent mean ± s.e.m. Different letters indicate statistically significant differences among treatments (One-Way ANOVA: A) for 6h *F_3-10_* = 4.195, *p* = 0.0366; for 14h *F_3-11_* = 21.48, *p* < 0.0001; for 24h *F_3-7_* = 5.206, *p* = 0.0334; for 48h *F_3-8_* = 1.328, *p* = 0.3314; B) for 6h *F_3-8_* = 9.595, *p* = 0.005; for 14h *F_3-8_* = 7.504, *p* = 0.0103; for 24h *F_3-8_* = 95.89, *p* < 0.0001; C) for 3h *F_3-8_* = 484.6, *p* < 0.0001; for 6h *F_3-8_* = 36.48, *p* < 0.0001; for 14h *F_3-8_* = 5.773, *p* = 0.0212; for 24h *F_3-8_* = 0.05704, *p* = 0.9809; D) for 3h *F_3-8_* = 96.41, *p* < 0.0001; for 6h *F_3-8_* = 134.8, *p* < 0.0001; for 14h *F_3-8_* = 441.2, *p* < 0.0001; for 24h *F_3-8_* = 38.96, *p* < 0.0001; for 48h *F_3-8_* = 73.55, *p* < 0.0001; E) for 3h *F_3-8_* = 119.4, *p* < 0.0001; for 6h *F_3-8_* = 111.5, *p* < 0.0001; for 14h *F_3-8_* = 648, *p* < 0.0001; for 24h *F_3-8_* = 303.3, *p* < 0.0001; for 48h *F_3-8_* = 392.5, *p* < 0.0001; F) for 3h *F_3-8_* = 1035, *p* < 0.0001; for 6h *F_3-8_* = 349.4, *p* < 0.0001; for 14h *F_3-8_* = 2179, *p* < 0.0001; for 24h *F_3-8_* = 674.3, *p* < 0.0001; G) for 3h *F_3-8_* = 688, *p* < 0.0001; for 6h *F_3-8_* = 382.7, *p* < 0.0001; for 14h *F_3-8_* = 240.5, *p* < 0.0001; for 24h *F_3-8_* = 251, *p* < 0.0001).


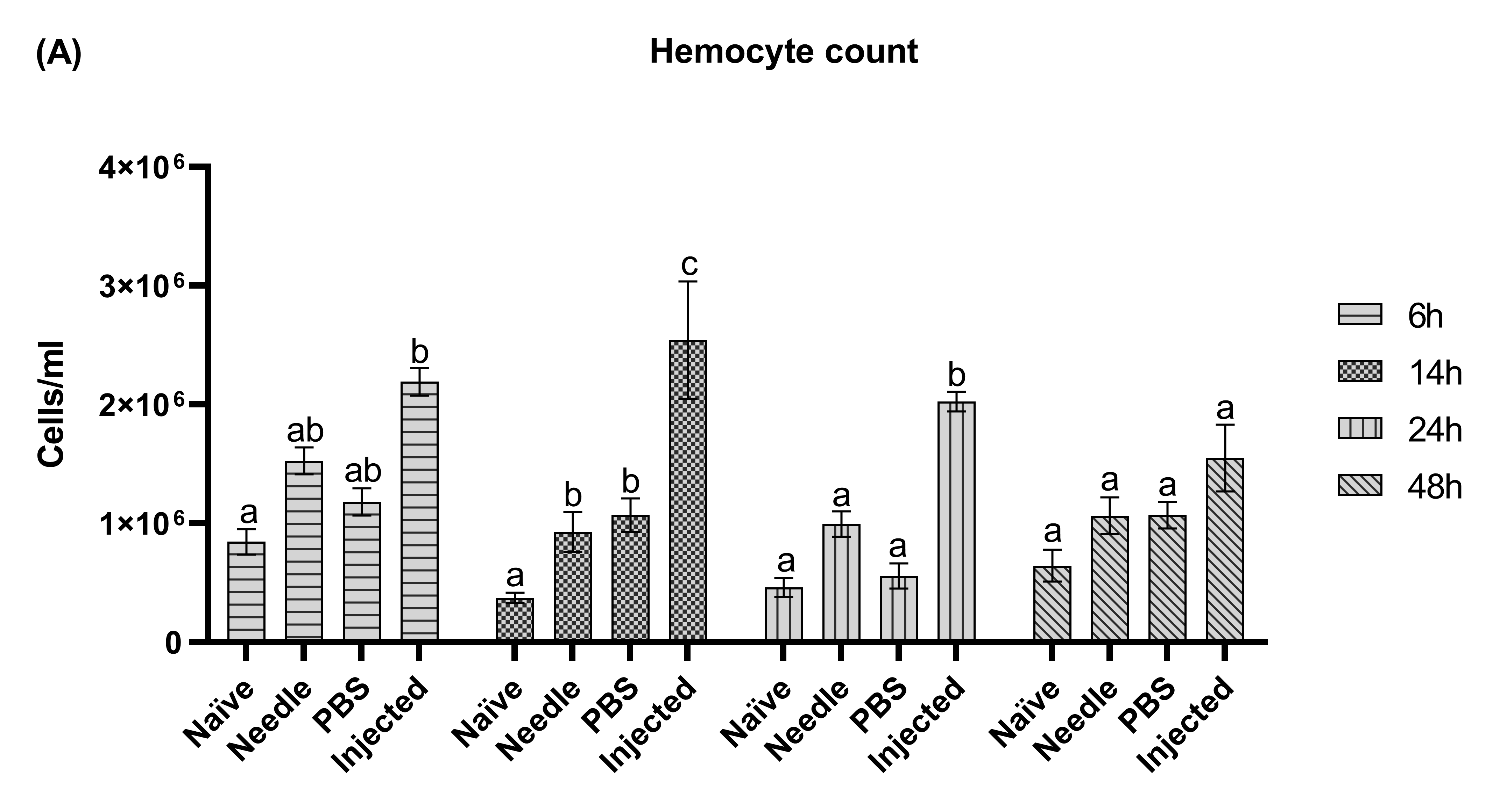


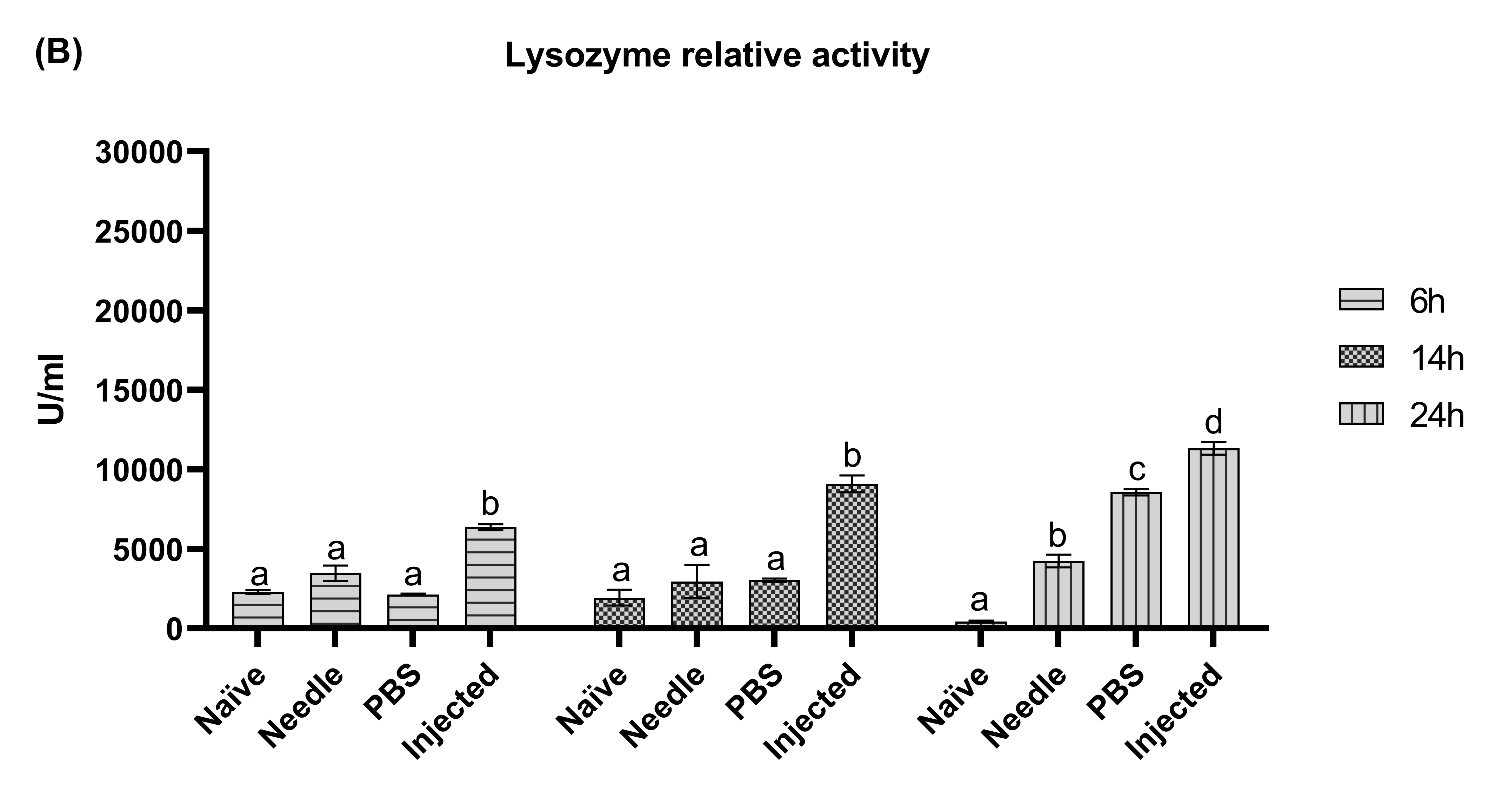

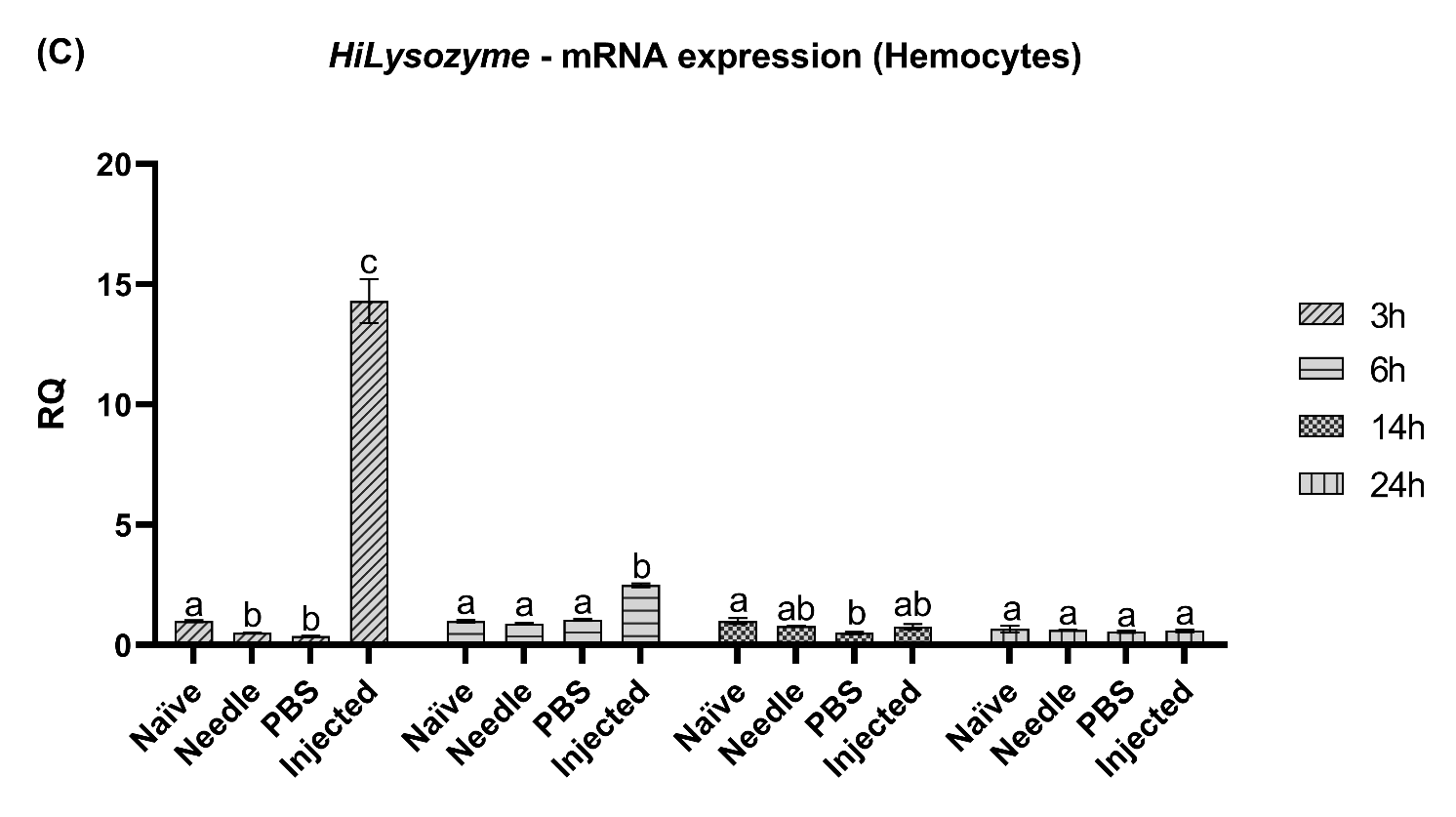

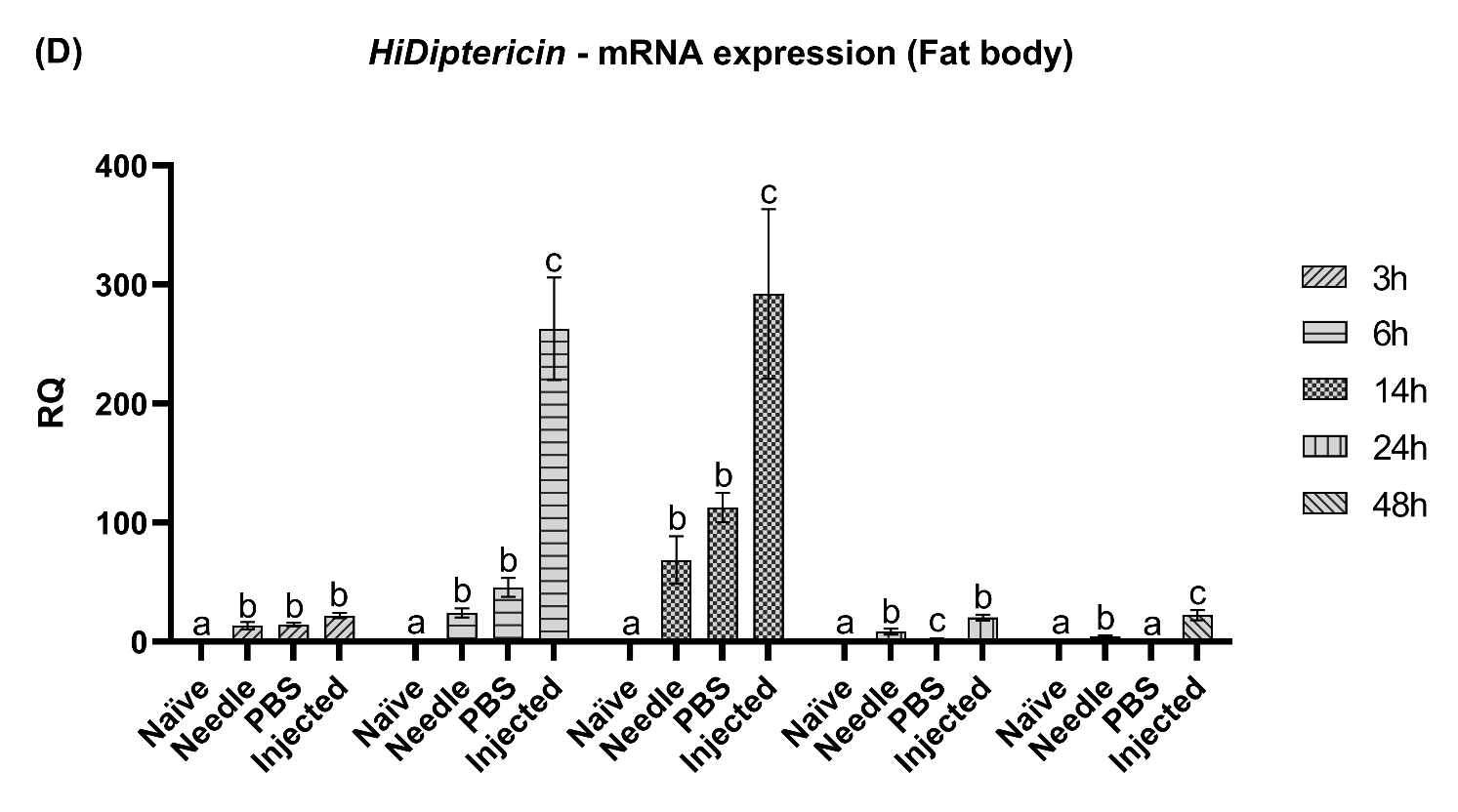

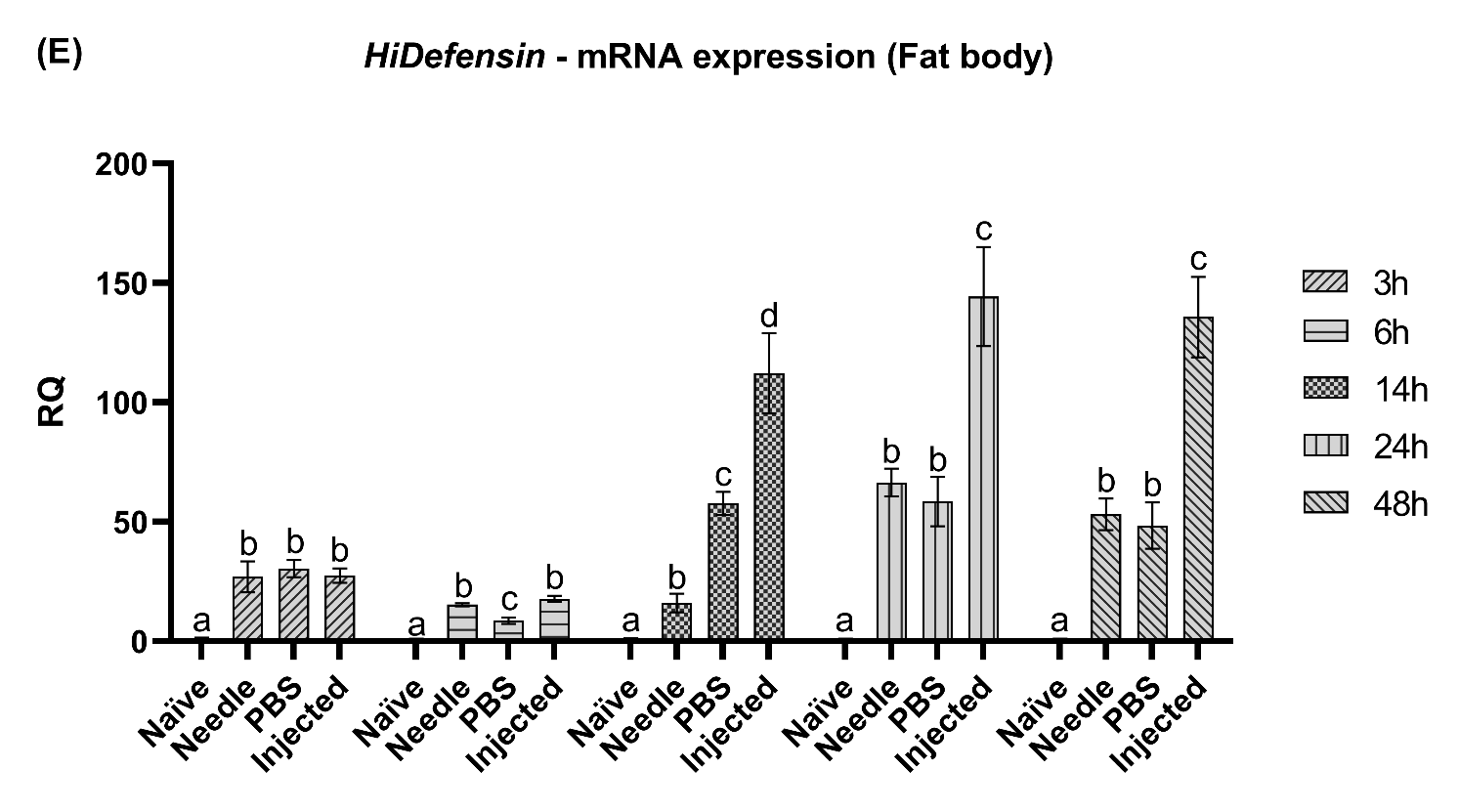

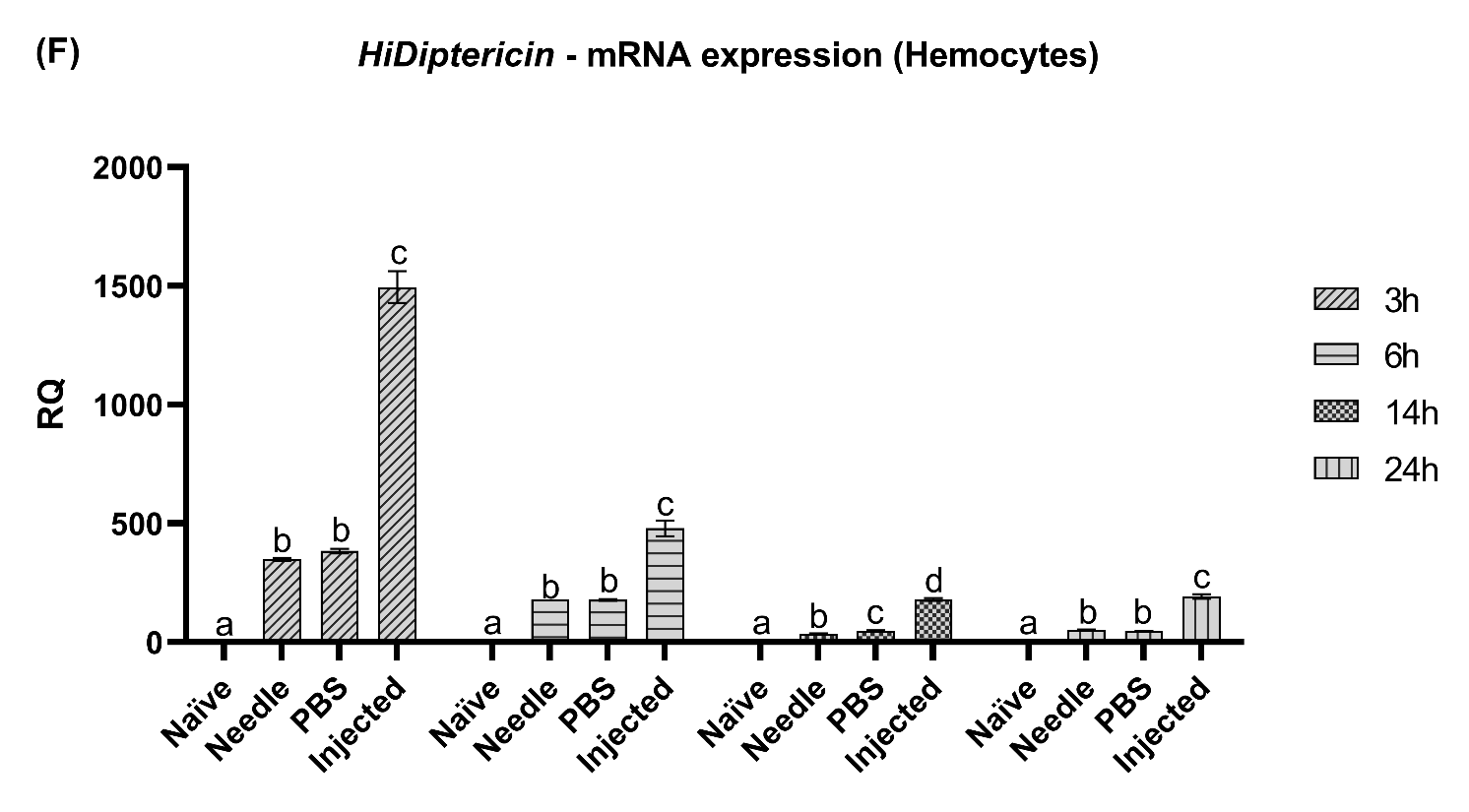

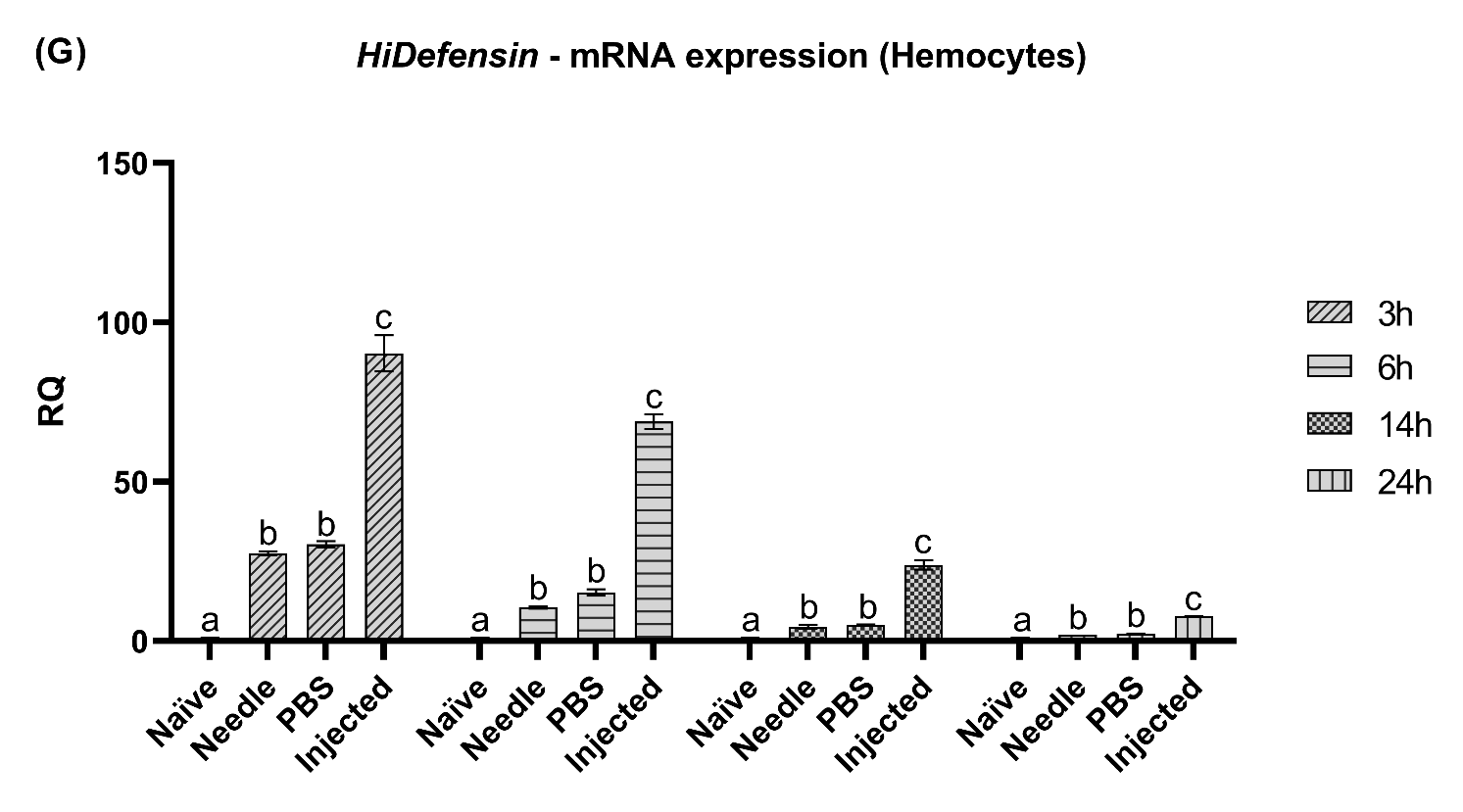


**Supplementary Figure 2 - Analysis of the immune response of naïve larvae being subjected to starvation at different time points.**

To verify the effects of starvation on naïve larvae over time, immune markers at different time points were analyzed. For this purpose, the number of hemocytes (A), lysozyme activity (B), *HiLysozyme* expression (C), *HiDiptericin* (D) and *HiDefensin* (F) expression in the fat body, *HiDiptericin* (E) and *HiDefensin* (G) expression in hemocytes were evaluated. Values represent mean ± s.e.m. Different letters indicate statistically significant differences among treatments (One-Way ANOVA: A) *F_3-10_* = 1.928, *p* = 0.189; B) *F_3-8_* = 2.695, *p* = 0.1166; C) *F_3-8_* = 0.02593, *p* = 0.9939; D) *F_4-10_* = 0.01799, *p* = 0.9992; E) *F_3-8_* = 0.02321, *p* = 0.9948; F) *F_4-10_* = 0.02753, *p* = 0.9983; G) *F_3-8_* = 0.001694, *p* = 0.9999).


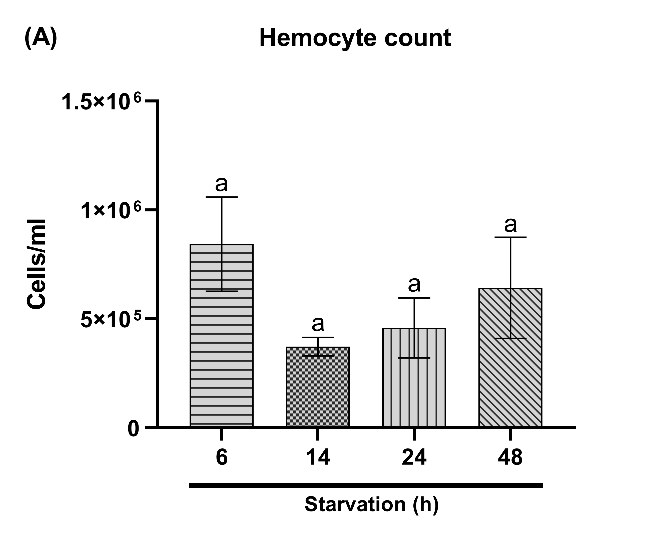


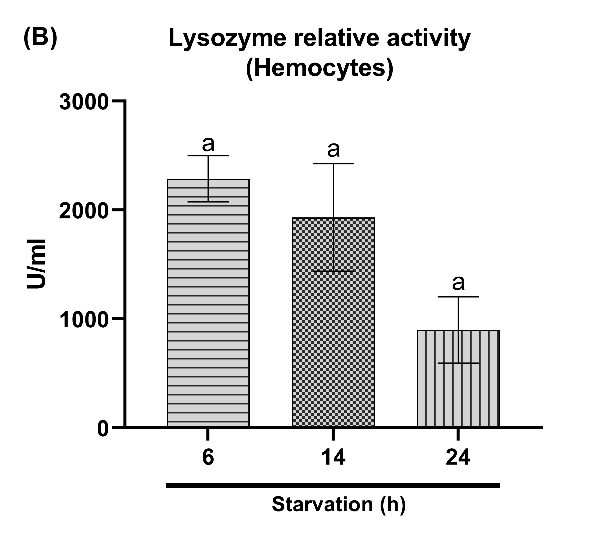

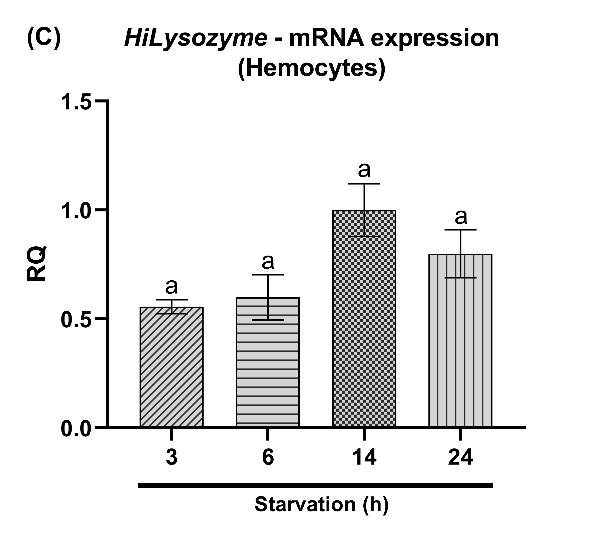


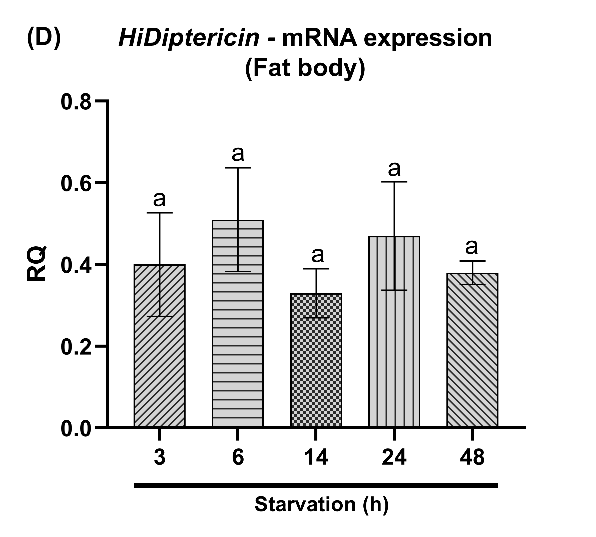

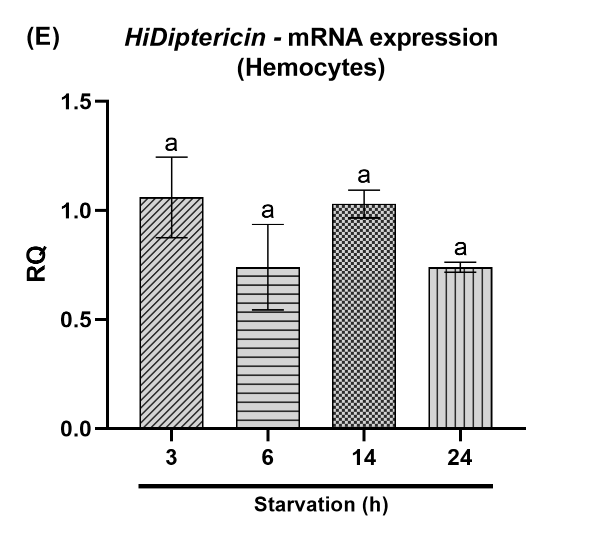

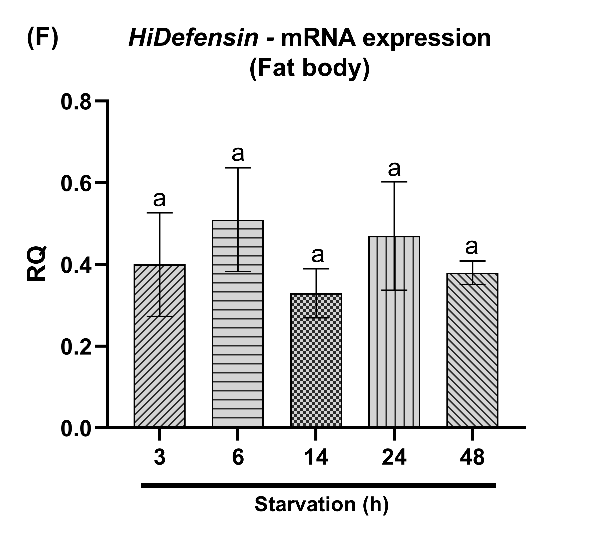

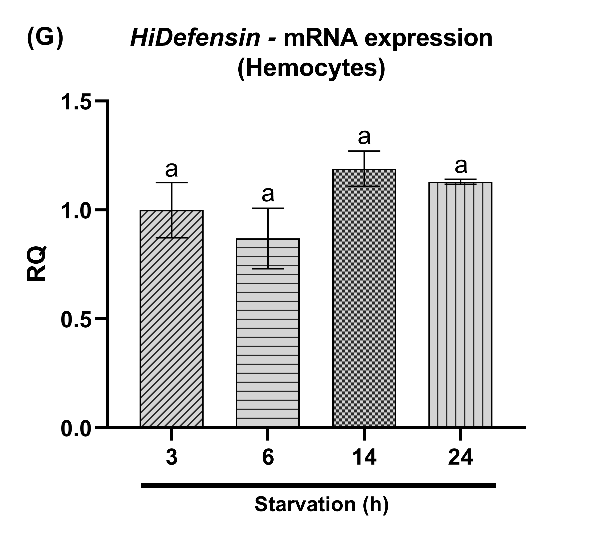


**Supplementary Figure 3. Survival rate of larvae infected with different concentrations of *E. coli/M. luteus* mix.**

To assess the effects of different concentrations of *E. coli/M. luteus* mix on the survival rate of *H. illucens* larvae, the injection of 10^4^, 10^5^, 10^6^, 10^7^, 10^8^, and 10^9^ CFU/ml bacteria was performed and the percentage of survived larvae was analyzed. The comparison of survival curves was performed by log-rank test (Chi-square = 88.85, *df* = 5, *p* < 0.0001).


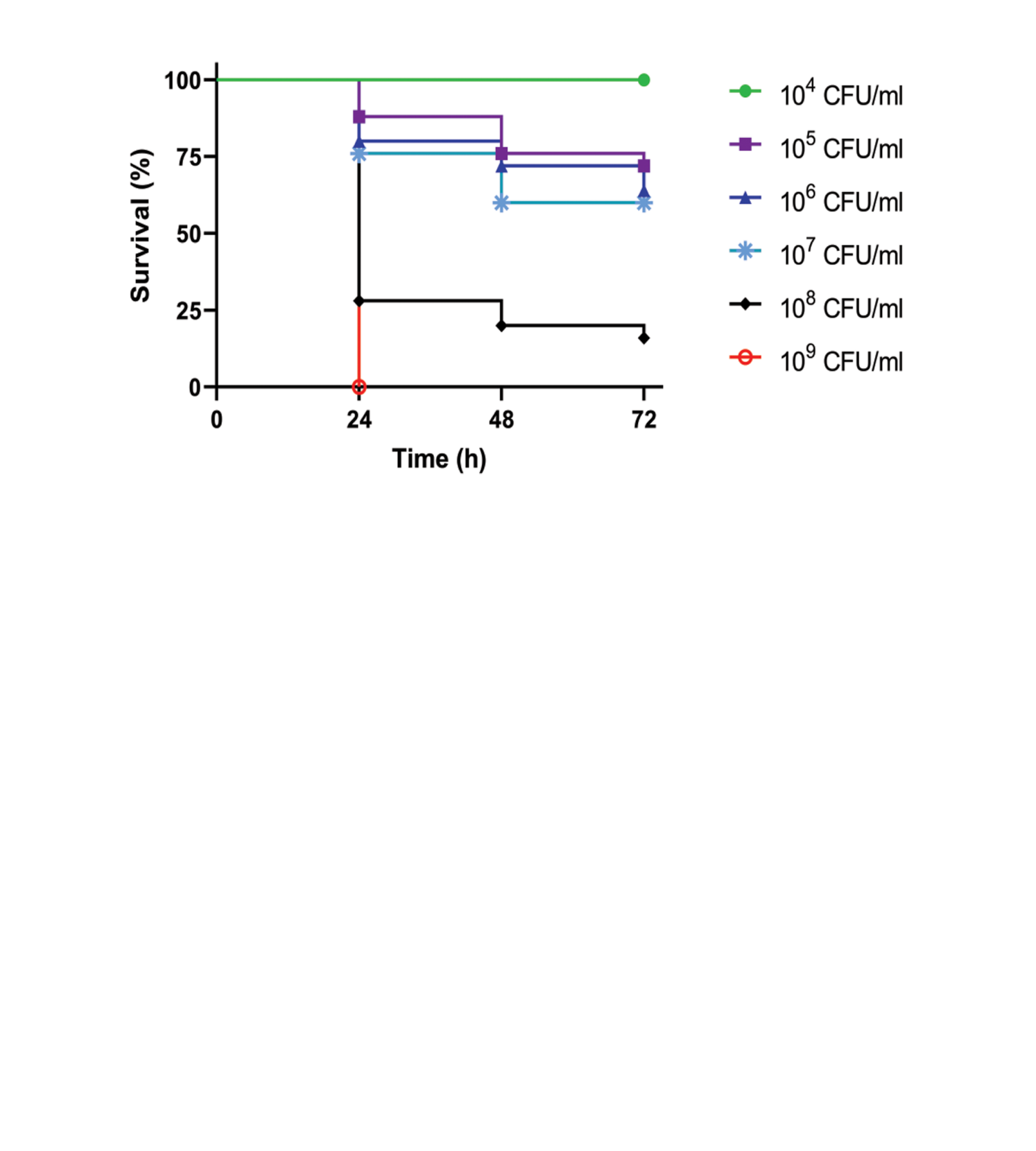

Supplement: Supplementary file 1 [file DataSheet_1.docx]
